# A Risk-Based Approach for Managing Aquaculture Used Oxytetracycline-Induced TetR in Surface Water Across Taiwan Regions

**DOI:** 10.3389/fphar.2021.803499

**Published:** 2021-12-23

**Authors:** Tien-Hsuan Lu, Chi-Yun Chen, Wei-Min Wang, Chung-Min Liao

**Affiliations:** Department of Bioenvironmental Systems Engineering, National Taiwan University, Taipei, Taiwan

**Keywords:** oxytetracycline, antibiotic resistance, pharmacodynamic, probabilistic risk assessment, pharmaceutical residues

## Abstract

Oxytetracycline (OTC), one of the most important antibiotics in aquaculture industry, has been linked to emergence of antibiotic resistant genes in the aquatic environment. Given rapid growth of the aquaculture industry and unregulated use of antibiotics, it is necessary to implement measures to mitigate the impact of antibiotic resistance risk on environmental and human health. However, there is a lack of quantitative models to properly assess risk of antibiotic resistance associated with environmentally relevant antibiotic residues. To address this issue, here we developed a computational framework to assess antibiotic resistance risk posed by low-concentration OTC in aquaculture ponds and rivers across Taiwan regions. To this end, estimated amount of aquaculture used OTC as a crucial input parameter was incorporated into a multimedia fugacity model to predict environmental concentrations of OTC in surface water/sediment. A pharmacodynamic-based dose–response model was used to characterize the OTC concentration–antibiotic resistance relationships. The risk of antibiotic resistance selection in an aquatic environment could be assessed based on a probabilistic risk model. We also established a control measure model to manage the risks of substantial OTC-induced antibiotic resistance impacts. We found that OTC residues were likely to pose a high risk of tetracycline resistance (tetR) genes selection in aquaculture ponds among all the study basins, whereas risk of tetR genes selection in rivers experienced a variably changing fashion. We also showed that it was extremely difficult to moderate the tetR genes selection rates to less than 10% increase in aquaculture ponds situated at northeastern river basins in that the minimum reductions on OTC emission rates during spring, summer, and autumn were greater than 90%. On the other hand, water concentrations of OTC during spring and summer in southwestern rivers should be prioritized to be severely limited by reducing 67 and 25% of OTC emission rate, respectively. Overall, incorporating a computational fugacity model into a risk assessment framework can identify relative higher risk regions to provide the risk-based control strategies for public health decision-making and development of robust quantitative methods to zero-in on environment with high risk of tetR genes selection in relation to aquaculture-used pharmaceutical residues.

## Introduction

Prophylactic use of antibiotics in aquaculture is highly likely to increase the risk of selection of antibiotic resistance (Chereau et al., 2017). If there are no collectors to catch uneaten medicated feed, antibiotics will be deposited in water and sediments after administration, leading to selection pressure on environmental bacteria to develop resistance and to facilitate horizontal gene transfer (HGT) between bacteria (Ashbolt et al., 2013; Cabello et al., 2016; Heuer et al., 2009). As a “genetic hotspot” for gene exchange, aquaculture can supply antibiotic resistance determinants to natural environments such as rivers which are considered reservoirs and a dissemination route for reintroduction of antibiotic resistance into humans (Singh et al., 2019; Kim and Cha, 2021). To address this emerging problem, legislative measures and plausible solutions should be suggested for antibiotic use policy, one of the high-profile concerns which have arisen with rapid growth of aquaculture industry (Caruso, 2016; Anderson et al., 2019).

Oxytetracycline (OTC), one of the most used antibiotics in aquaculture, is indicated to treat fish bacterial diseases, including furunculosis, aeromonosis, pseudomonosis, lactococcosis, and vibriosis, via feed, bath treatment, and injection (Leal et al., 2019). In total, 73% of the top 15 aquaculture-producing countries applied OTC during the period 2008–2018 (Lulijwa et al., 2019). OTC was classified into the category of Veterinary Critically Important Antimicrobial Agents in which particular attention should be paid (OIE World Organisation for Animal Health, 2019). Moreover, the use of OTC was verified to be associated with occurrence of tetracycline resistance (tetR) genes in aquaculture facilities (Seyfried et al., 2010). Due to the inherent connections between aquaculture area with open water bodies, fish ponds are coined reservoirs of antibiotic resistance genes (ARGs), suggesting that OTC treatment in aquaculture facilities and farms may favor the process of HGT to introduce tetR genes to the environment (Lulijwa et al., 2019; Seyfried et al., 2010). It was also found that the occurrence of tetR genes in rivers could be attributed to the usage of antibiotics in riverside aquaculture farms (Harnisz et al., 2015).

Recently, concentrations of antibiotics in air, water, soil, and sediment compartments in river basins have been successfully estimated by applying the fugacity model, one of the most successful types of multimedia environmental models, informing estimates for ecological and human health risk assessment and linking to bacterial resistance (Zhang et al., 2015; Chen et al., 2018; Dong et al., 2019). The fugacity approach was first established in Mackay (1979). The ability to define characteristics for a variety of media and processes such as advection, reaction, and intermedia transport enables us to set up mass balance equations and then deduce fugacities, concentrations, fluxes, and amounts (Mackay, 2001; Parnis and Mackay, 2021). Due to the simplicity in structure and accessible in parameterization, the fugacity models are prevailing and commonly used and have been widely applied to predict the behavior of chemicals in either terrestrial or aquatic ecosystems, ranging from regional to global scales (Kong et al., 2016; Su et al., 2019).

Although OTC is approved to be used in aquaculture and is also identified in aquatic environments in Taiwan, including aquaculture ponds and rivers (Lai et al., 2018; Lin et al., 2008; Lin and Lin, 2008; 2009; 2010), there is a lack of systematic study investigating the impact of aquaculture used OTC on the selection of tetR genes in pond water as well as river water that would be influenced by the discharges from aquaculture. As a result, to provide decision-support for government and managers to prevent overuse of OTC in aquaculture situated at river basins across Taiwan region, the goals of this study are fourfold: (i) to estimate the monthly amount of OTC used in aquaculture, (ii) to estimate the concentrations of OTC in water of aquaculture ponds and rivers by applying a fugacity model, (iii) to construct the dose–response relationship between OTC concentration and antibiotic resistances, and (iv) to assess the risk of selection of tetR genes in a water environment due to residual OTC used by aquaculture.

## Materials and Methods

### Study Framework

**FIGURE 1:**
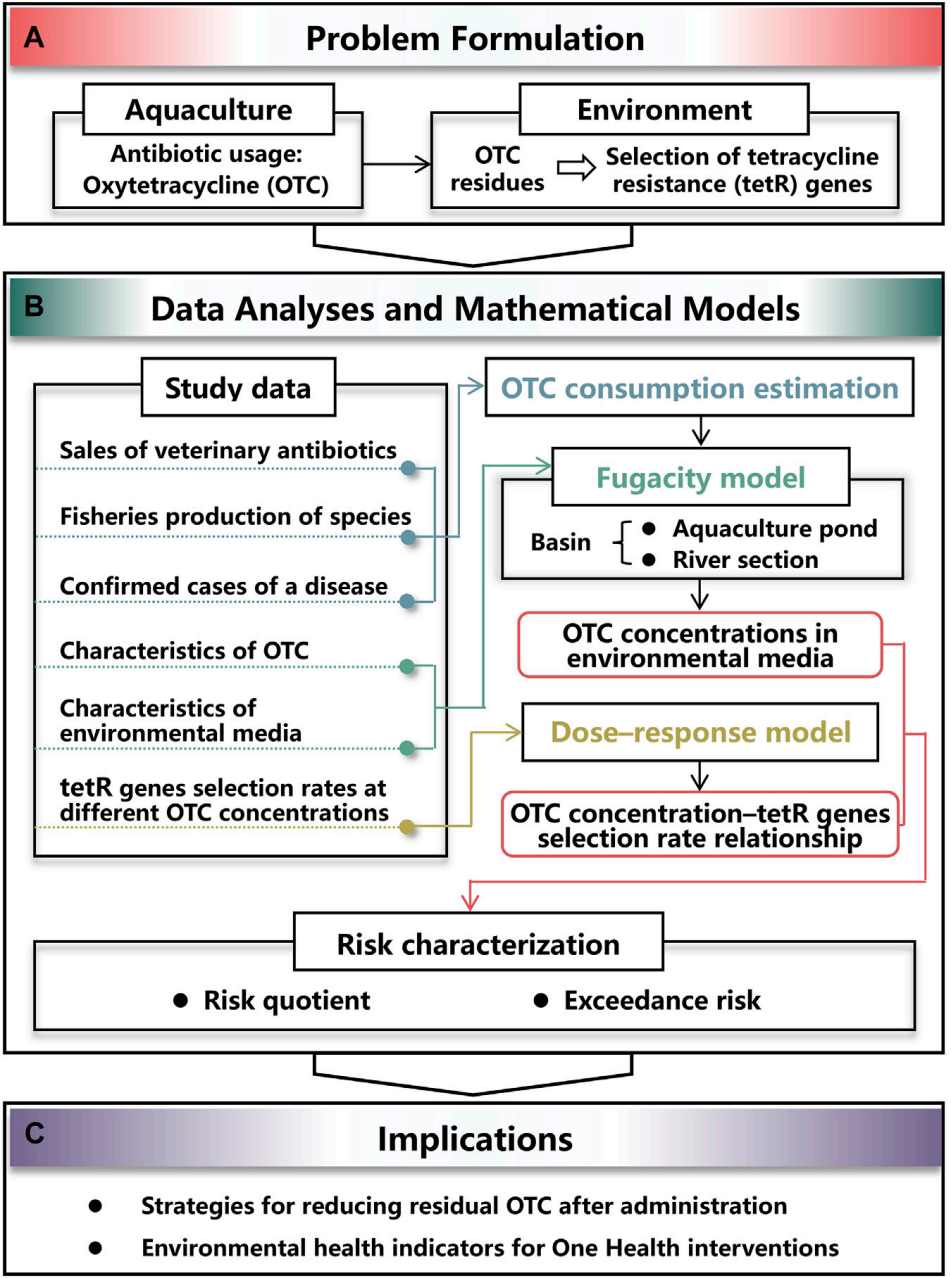
Schematic illustrating the study framework constituted by **(A)** problem formulation of resistance selection induced by residual oxytetracycline (OTC) used in aquaculture, **(B)** process of assessing the risk by analyzing study data and applying mathematical models, and **(C)** implications for interventions.

To explore the effect of aquaculture used OTC residues on selection of tetR genes in environment (Figure 1A), first, data were collected and collated for the following purposes of data analyses (Figure 1B): (i) OTC consumption was estimated based on sales of veterinary antibiotics, fisheries production of species, and confirmed cases of a disease (Figure 1B), (ii) OTC concentrations in aquaculture ponds and river section were estimated by applying the developed fugacity model in which characteristics of OTC and environmental media were considered (Figure 1B), and (iii) the appropriated dose–response model was used to construct the OTC concentration–tetR genes selection rate relationship (Figure 1B). As a result, the risk of tetR genes selection could be characterized based on OTC concentration estimates and the dose–response relationship (Figure 1B). Finally, quantitative results could provide strategies for reducing residual OTC and be used as environmental health indicators for One Health-based interventions (Figure 1C).

### Study Data: OTC Emission Estimation

Amount of OTC consumed in aquaculture cannot be directly accessed; however, annual sales of veterinary antibiotics in Taiwan during the period 2015–2019 are available from Bureau of Animal and Plant Health Inspection and Quarantine (BAPHIQ), Taiwan (BAPHIQ Bureau of Animal and Plant Health Inspection and Quarantine, Council of Agriculture, Executive Yuan, 2021). Indeed, conditions of OTC administration are dependent on several factors such as nature and extension of disease, and species, age, and condition of the fish (Leal et al., 2019). To reasonably employ the available data to estimate amount of OTC emitted from aquaculture farms in each basin, production- and cases-based estimations were conducted.

Briefly, the product of annual sales of veterinary antibiotics (*M*_VET-AN_) and percentage of OTC among veterinary antibiotics (*P*_OTC_) was calculated to obtain annual amount of OTC used in aquaculture (*M*_OTC-AN_) (Supplementary Figure 1A). Then, amount of OTC used in aquaculture in region *i* (*M*_OTC-A*i*_) was estimated based on the annual fisheries production data that were adopted from FACOA Fisheries Agency, Council of Agriculture, Executive Yuan (2021) (Supplementary Figures 1B,C). In this stage, only the production of species that are approved to be treated with OTC was included to estimate the potential proportion of OTC applied by region *i* (*P*_AP-*i*_) (Supplementary Figure 1B).

The top five regions with major production of species that are approved to be treated with OTC were selected in this study. For each selected region, the potential proportion of annual OTC applied in each month (*P*_OTC-M*i*_) was calculated based on monthly confirmed cases of motile aeromonad disease that were adopted from BAPHIQ Bureau of Animal and Plant Health Inspection and Quarantine, Council of Agriculture, Executive Yuan (2021) to estimate the amount of OTC used in different month (*M*_OTC-M*i*_) (Supplementary Figures 1D,E). Basins in the top five regions with major production of species that are approved to be treated with OTC were selected. Finally, the region-based amount was converted to monthly amount of OTC used in aquaculture in river basin *k* (*M*_OTC-M*k*_) by considering the proportion of aquaculture area in a region that is adjacent to a basin (*P*_A*i*-B*k*_) (Supplementary Figures 1F,G).

Species that are approved to be treated with OTC in Taiwan are classified into seven orders: Anguilliformes, Gonorynchiformes, Perciformes, Salmoniformes, Decapoda, Anura, and Testudines in the Guidelines for the Use of Drugs in Animals (Law & Regulations Database of The Republic of China, 2019). The top five regions with fisheries productions of these species were mainly situated in the southwestern area (Kaohsiung City, Tainan City, Pingtung County, Chiayi County) with only one at the northeastern area (Ilan County) (Supplementary Figure 2). Order Perciformes occupied a large proportion of the production, followed by Gonorynchiformes and Decapoda (Supplementary Figure 2). The mean potential proportion of OTC applied by Kaohsiung City was 0.20, which was the highest, followed by 0.18 in Tainan City and Pingtung County, and 0.14 in Chiayi County and Ilan County (Supplementary Table 1). On the other hand, aquaculture sectors situated at northeast (Lanyang river basin) and southwest (Potzu, Tsengwen, Yenshui, Agongdian, Kaoping, and Tungkang river basins) were therefore selected to estimate their emissions of OTC based on the collected information on the confirmed cases of motile aeromonad disease, one of the fish bacterial diseases that are approved to be treated with OTC in Taiwan, and the distribution of aquaculture area (Supplementary Figure 2, Supplementary Table 1).

### Study Data: Dose–Response Data

It has been found that sub-lethal antibiotic concentrations pose selective pressures for bacteria to develop resistances (Gullberg et al., 2001; Khan et al., 2017). Knapp et al. (2008) evidenced that OTC exposure at low levels could contribute to selection of tetR genes in aquatic systems. They performed experiments by adding OTC at the concentrations of 0, 5, 20, 50, and 250 μg L^−1^ in mesocosms that contained water without previous direct exposure to antibiotics. The OTC concentrations were regularly analyzed. The tetR genes, including *tet*(B), *tet*(L), *tet*(M), *tet*(O), *tet*(Q), and *tet*(W), 16S-rRNA genes, and regulatory gene for the Tn916/Tn1545 transposon family were quantified. Furthermore, they calculated the selection rates of tetR genes and transposon at different levels of OTC concentrations (Supplementary Table 2). Those data were used in the dose–response analysis to quantify the effect of OTC concentration on selection of tetracycline resistance in surface water.

### Fugacity Model

**FIGURE 2:**
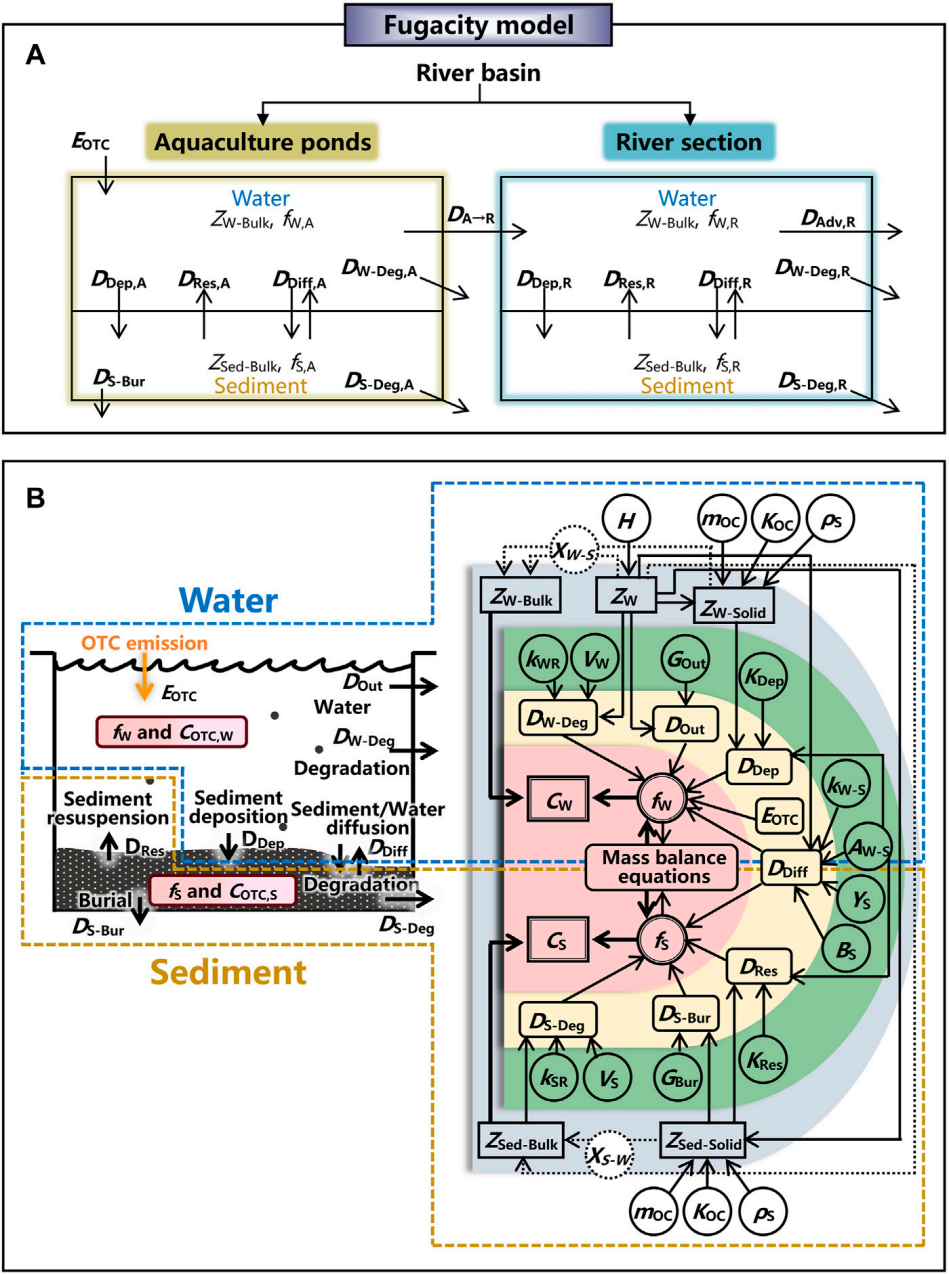
Schematic illustrates **(A)** the fugacity model describing the fate of OTC emitted from aquaculture to a river section within a basin and **(B)** the process of estimating the concentrations of OTC in water and sediment by the fugacity model. Symbol meanings: *E*_OTC_ is the emission rate of OTC, *Z*_W-Bulk_ and *Z*_Sed-Bulk_ are the bulk fugacity capacities of water and sediment, respectively, *Z*_W_, *Z*_W-Solid_, and *Z*_Sed-Solid_ are the fugacity capacities of water, particles in water, and particles in sediment, respectively, *f*_*i*_ is the fugacity, *D*_Dep_, *D*_Res_, *D*_Diff_, *D*_W-Deg_, *D*_S-Deg_, *D*_S-Bur_, *D*_A→R_, and *D*_Adv,R_ are the transport parameters for the processes of sediment deposition, sediment resuspension, sediment/water diffusion, degradation in water, degradation in sediment, sediment burial, discharge from aquaculture ponds, and river discharge, respectively, *C*_OTC,*i*_ is the concentration of OTC, subscripts W, S, A, and R represent the values for water, sediment, aquaculture ponds, and a river section, respectively, *H* is the Henry’s law constant, *m*_OC_ is the mass fraction organic carbon, *K*_OC_ is the organic carbon–water partition ratio, *ρ*_S_ is the density of solid, *X*_W-S_ is the volume fraction of particles in water, *X*_S-W_ is the volume fraction of water in sediment, *k*_WR_ is the degradation rate in water, *k*_SR_ is the degradation rate in sediment, *V*_W_ is the volume of water, *V*_S_ is the volume of sediment, *G*_Out_ is the water outflow rate, *G*_Bur_ is the burial rate, *K*_Dep_ is the sediment deposition rate, *K*_Res_ is the sediment resuspension rate, *k*_W-S_ is the water-side mass transfer coefficient over sediment, *Y*_S_ is the diffusion path lengths in sediment, and *B*_S_ is the molecular diffusivity in sediment.

A level III fugacity model was constructed to describe the fate of OTC used in aquaculture within a basin. A river section could be affected by the discharges from aquaculture ponds. Thus, for a river basin, two sub-environments were included, i.e., aquaculture ponds and a river section. In each sub-environment, the environment was divided into water and sediment compartments (Figure 2A).

The process of estimating OTC concentrations in water and sediment by the fugacity model is illustrated in Figure 2B. Based on the algorithm of the fugacity model, environmental concentrations of OTC in bulks of water (*C*_W_, mol m^−3^) and sediment (*C*_S_, mol m^−3^) of aquaculture ponds and a river section could be obtained by multiplying the fugacity (*f*) and the fugacity capacity (*Z*) (the detailed mathematical manipulation is performed in Supplementary Material).

$$C_{\text{W},\text{A}}=Z_{\text{W}-\text{Bulk}}f_{\text{W},\text{A}},$$ (1)

$$C_{\text{S},\text{A}}=Z_{\text{S}-\text{Bulk}}f_{\text{S},\text{A}},$$ (2)

$$C_{\text{W},\text{R}}=Z_{\text{W}-\text{Bulk}}f_{\text{W},\text{R}},$$ (3)

$$C_{\text{S},\text{R}}=Z_{\text{S}-\text{Bulk}}f_{\text{S},\text{R}},$$ (4)

where *Z*_W-Bulk_ and *Z*_S-Bulk_ are the bulk fugacity capacities of water and sediment (mol Pa^−1^ m^−3^), respectively; *f*_W_ and *f*_S_ are the fugacities for the bulk of water and sediment (Pa), respectively, and the subscripts A and R present the aquaculture pond and river basin, respectively.

In this study, the concentrations of OTC in water of aquaculture ponds and river sections within Lanyang, Potzu, Tsengwen, Yenshui, Agongdian, Kaoping, and Tungkang river basins were estimated based on the fugacity model. To estimate the concentration of OTC in environment, fugacity capacities (*Z*s) and fugacities (*f*s) should be calculated. The equations and parameters used for calculating Z-values for environmental media are listed in Supplementary Tables 3,4, respectively. To calculate fugacities, emission rate (*E*_OTC_) and transport parameters (D-values) were essential. *E*_OTC_s were estimated based on the process illustrated in Supplementary Figure 1, whereas the equations for calculating D-values are listed in Supplementary Table 5. Some parameters such as area of water phase and discharge flow rate used in the calculation of D-values varied depending on the characteristics of aquaculture ponds and river sections for river basins. Therefore, parameters used for D-value calculation in the same values among river basins are listed in Supplementary Table 6, whereas those in the different values depending on the month and river basins are listed in Supplementary Table 7.

### Dose–Response Analysis

To investigate the effect of OTC residues on antibiotic resistance in environment, the dose–response relationships between OTC concentration and tetR genes and transposon selection rates were constructed. Benchmark dose (BMD) approach which is applicable to ecological risk assessment and dose–response modeling of health effect (U.S. EPA U.S. Environmental Protection Agency, 2012) was applied to suggest an acceptable level of tetR genes selection rate. Benchmark concentrations (BMCs) corresponding to 10% increase in the benchmark response (*BMR*10) (*BMC*10) was determined based on the dose–response relationships.

### Risk Characterization

Risk of tetR genes selection in the environment can be used as an indicator for environmental health. In the Bayesian inference-based probabilistic risk model, the prior probabilities of OTC concentration in the water [*P*(*C*_W_)] can be multiplied with the conditional probabilities *P*(*r*_ARG_|*C*_W_) based on the dose–response model constructed to describe the relationship between OTC concentration and tetR genes selection rate (*r*_ARG_), resulting in a posterior probability *Φ*(*R*_ARG_) that can be expressed mathematically as

$$\Phi \left(R_{\text{ARG}}\right)=P\left(C_{\text{W}}\right)P\left(r_{\text{ARG}}\text{|}C_{\text{W}}\right),$$ (5)

where *Φ*(*R*_ARG_) indicates the risk of tetR genes selection in environment. The exceedance risk (*ER*) can then be expressed as 1 − *Φ*(*R*_ARG_).

On the other hand, to assess the potentially environmental risk, risk quotient (*RQ*) of increasing tetR genes selection rate can be calculated as

$$RQ=\frac{C_{\text{W}}}{BMC10},$$ (6)

where *C*_W_ is the environmental OTC concentration in water of aquaculture ponds or a river section estimated by the fugacity model (μg L^−1^). *RQ* > 1, 0.1 ≤ *RQ* < 1, and *RQ* < 0.1, respectively, are corresponding to high, moderate, and low risks (Hanna et al., 2018).

### Control Measure Model

Based on the constructed fugacity model, it could be found that fugacities of water environment (*f*_W,A_ and *f*_W,R_) were proportional to OTC emission rate (*E*_OTC_) (Supplementary Eqs S11 and S13). Moreover, OTC concentrations in water environment of aquaculture ponds (*C*_W,A_) and a river section (*C*_W,R_) were proportional to *f*_W,A_ and *f*_W,R_, respectively (Eqs 1 and 3). Therefore, *C*_W,A_ and *C*_W,R_ were proportional to *E*_OTC_. A control measure model could be developed based on the proportional relationship among OTC concentration, fugacity, and emission rate and the constructed dose–response relationship between OTC concentration and tetR genes selection rate.

### Uncertainty Analysis

TableCurve 2D (Version 5.01, AISN software, Mapleton, OR, United States) was employed to perform the model fitting to construct the dose–response relationships. A Monte Carlo (MC) technique was performed with 10,000 iterations to obtain 2.5th and 97.5th %tiles as the 95% confidence interval (CI) or normal (N) and lognormal (LN) distributions for parameters and estimates. The Oracle^®^ Crystal Ball software (Version 11.1.2.4, Oracle Corporation, Redwood Shores, CA, United States) was used to implement the MC simulation.

## Results

### Emission Rate and OTC Concentration Estimates

**FIGURE 3:**
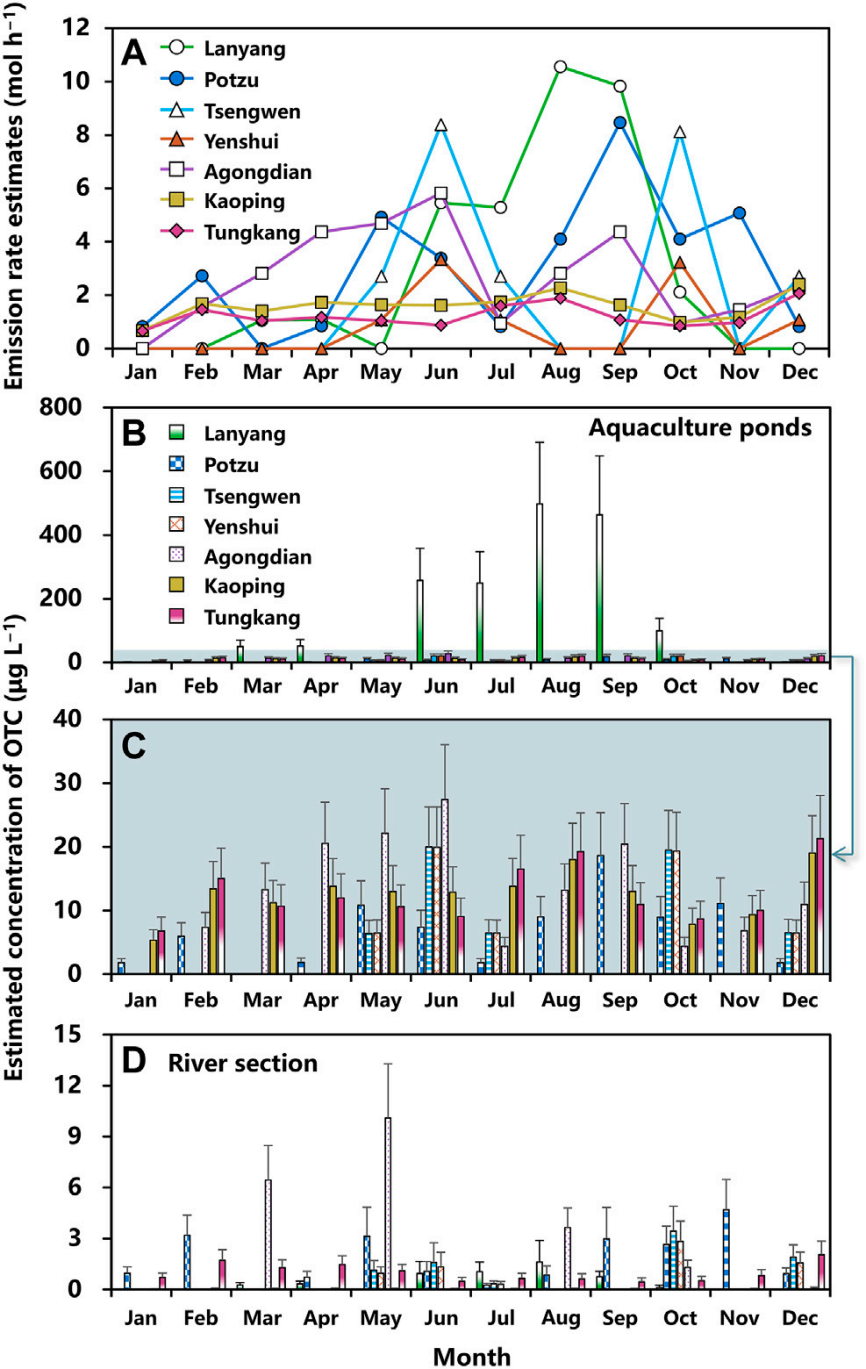
**(A)** Estimated monthly mean emission rates of OTC (mol h^−1^) for aquaculture situated at Layang, Potzu, Tsengweng, Yenshui, Agongdian, Kaoping, and Tungkang river basins. Monthly concentrations of OTC (μg L^−1^) in water of **(B,C)** aquaculture ponds and **(D)** a river section that affected by discharges from aquaculture situated at Layang, Potzu, Tsengweng, Yenshui, Agongdian, Kaoping, and Tungkang river basins. Error bar indicates standard deviation.

Estimated monthly averaged emission rates of OTC used in aquaculture sector situated at seven river basins during the period 2015–2019 are shown in Figure 3A. Excluding the null estimates, mean emission rates averaged over this 5-year interval in Lanyang (1.06–10.57 mol h^−1^), Potzu (0.82–8.47 mol h^−1^), Tsengwen (2.71–8.39 mol h^−1^), Yenshui (1.08–3.34 mol h^−1^), Agongdian (0.94–5.82 mol h^−1^), Kaoping (0.67–2.40 mol h^−1^), and Tungkang (0.66–2.07 mol h^−1^) river basins could be estimated (Figure 3A; Supplementary Table 8). The highest emission rate of OTC was 10.57 ± 3.59 (Mean ± SD) mol h^−1^ in Lanyang river basin in August, followed by 9.83 ± 3.34 mol h^−1^ in September, and 8.47 ± 2.93 in Potzu river basin in September (Figure 3A). The emission rates in Kaoping and Tungkang river basins varied within a narrow range yet shown in all months, demonstrating different patterns from other basins (Figure 3A).

Based on the estimated emission rates, monthly concentrations of OTC in aquaculture ponds and a river section in each basin could be estimated by applying the fugacity model (Figures 3B–D; Supplementary Table 9). Results showed that mean concentrations of OTC in water of aquaculture ponds of seven river basins ranged from 50.17 to 497.93 (Lanyang), 1.80–18.62 (Potzu), 6.42–19.98 (Tsengwen), 6.46–19.93 (Yenshui), 4.41–27.39 (Agongdian), 5.33–19.04 (Kaoping), and 6.81–21.31 (Tungkang) μg L^−1^ (Figures 3B,C). We found that the OTC concentrations in aquaculture ponds situated in Lanyang river basin were much higher than those in other basins with the highest concentration of 497.93 ± 193.03 μg L^−1^ in August, followed by 463.76 ± 184.38 μg L^−1^ in September, and 257.63 ± 100.79 μg L^−1^ in June (Figure 3B).

The mean concentrations of OTC in water of seven river sections within each river basins ranged from 0.13–1.62 (Lanyang), 0.25–4.70 (Potzu), 0.34–3.45 (Tsengwen), 0.30–2.84 (Yenshui), 1.31–10.10 (Agongdian), 0.03–0.11 (Kaoping), and 0.45–2.05 (Tungkang) μg L^−1^ (Figure 3D). The first and second highest concentrations of OTC both appeared in Agongdian River with estimates of 10.10 ± 3.19 and 6.44 ± 2.04 μg L^−1^ in May and March, respectively, followed by 4.70 ± 1.79 μg L^−1^ in Potzu River in November (Figure 3D).

### OTC Concentration-Dependent Resistance Selection

**FIGURE 4:**
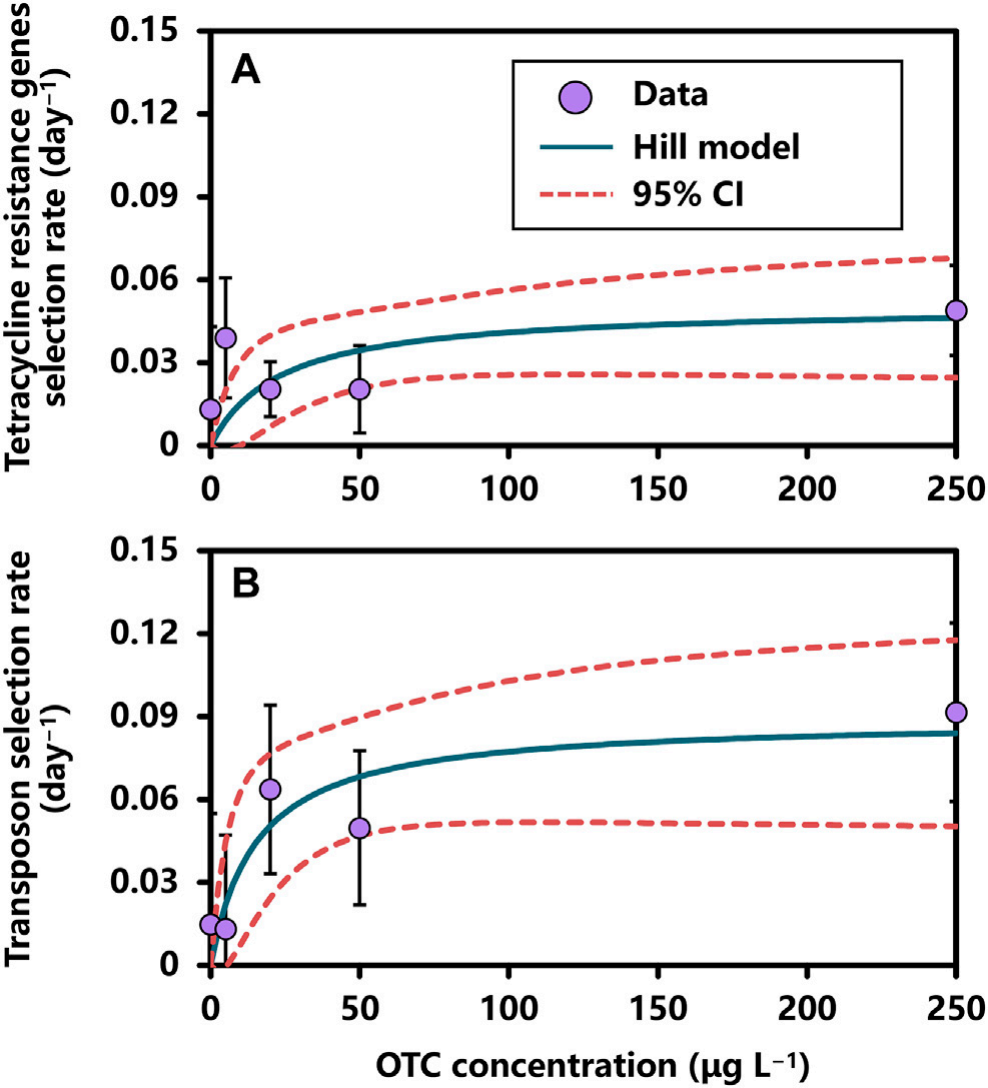
The relationships between OTC concentration and selection rates of **(A)** tetracycline resistance genes and **(B)** transposon described by the Hill-1 model of Michaelis-Menten function. Error bar indicates standard deviation.

The reconstructed dose–response relationships between (i) OTC concentration and tetR genes selection rate and (ii) OTC concentration and transposon selection rate could be fairly described by a Hill-1 model expressed as a Michaelis-Menten function (Figure 4; Supplementary Table 10).

Our results showed that *EC*50 estimates were 23.27 ± 21.39 (mean ± SE) in the OTC concentration–tetR genes selection rate relationship and 15.34 ± 12.74 in the OTC concentration–transposon selection rate relationship (Supplementary Table 10). The *r*_max_ estimates were both significant in OTC concentration–tetR genes selection rate (0.05 ± 0.01 days^−1^; *p*-value < 0.01) and OTC concentration–transposon selection rate (0.09 ± 0.02 days^−1^; *p*-value < 0.001) relationships. On the other hand, *BMC*10s for tetR genes and transposon selection rates were 2.59 μg L^−1^ (95% CI: 0.97–4.21) and 1.71 μg L^−1^ (95% CI: 0.67–2.75), respectively (Supplementary Table 10).

### Risk Estimates for Resistance Selection

**FIGURE 5:**
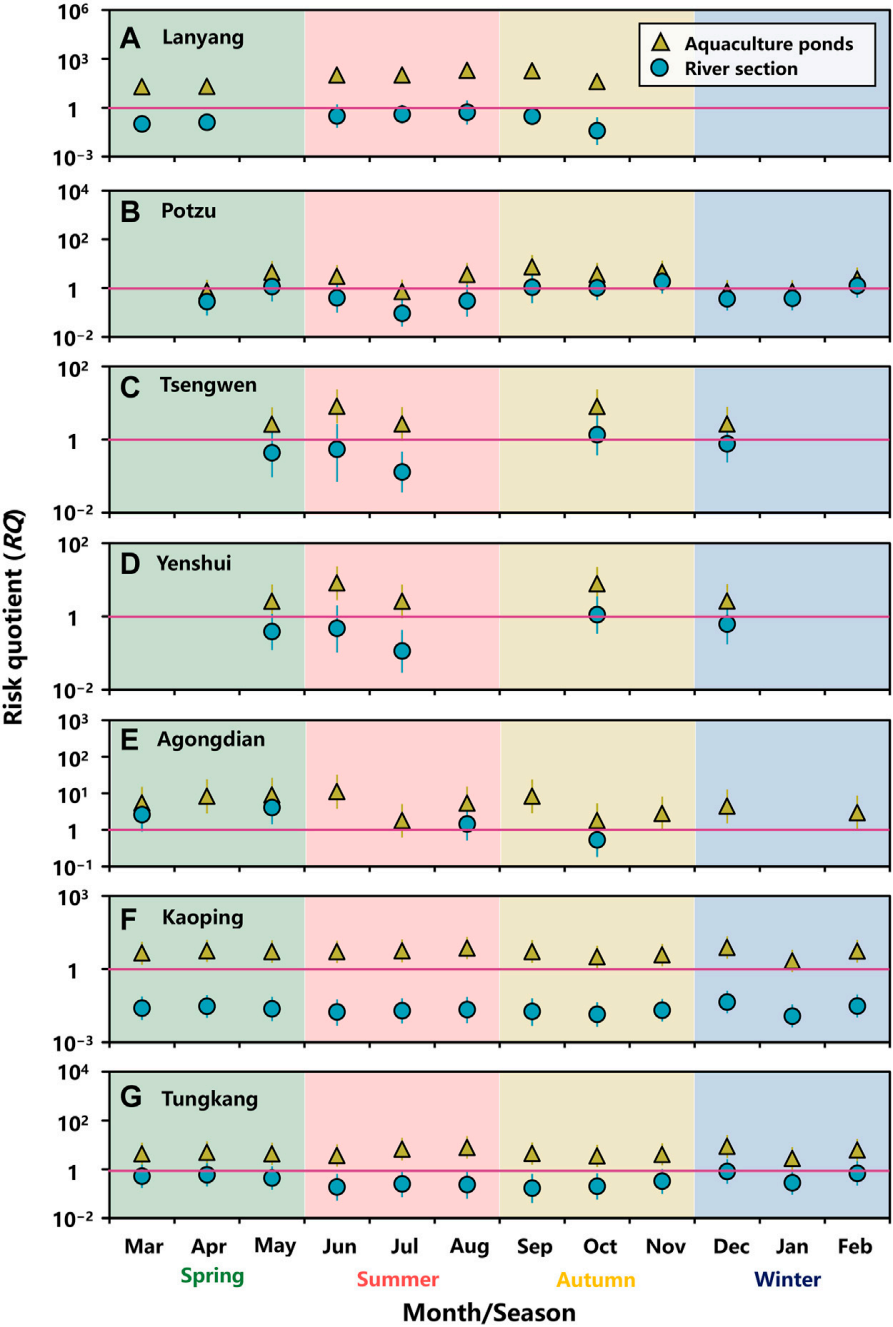
Monthly risk quotients (*RQ*s) for tetracycline genes selection in water environment of aquaculture ponds and a river section within **(A)** Lanyang, **(B)** Potzu, **(C)** Tsengwen, **(D)** Yenshui, **(E)** Agongdian, **(F)** Kaoping, and **(G)** Tungkang river basins. (Symbol: median, error bar: 2.5–97.5%-tile).

Monthly *RQ*s for tetR genes selection in water environment of aquaculture ponds and river sections subject to OTC residues were estimated (Figure 5; Supplementary Table 11). In aquaculture ponds, almost all the median *RQ*s were higher than 1, indicating that OTC residues were highly likely to cause tetR genes selection (Figure 5). Although the median *RQ*s for aquaculture ponds within Potzu river basin were lower than 1 in April, July, December, and January, the 75th %tile *RQ*s were higher than 1 (Figure 5B).

Our results showed that the risks of tetR genes selection in river sections could be variably changed in a high (*RQ* > 1), moderate (0.1 ≤ *RQ* < 1), and low (*RQ* < 0.1) fashion (Figure 5). Median *RQ*s for the river section within Lanyang river basin showed moderate risk of tetR genes selection, except for that in October with a low risk (Figure 5A). For Potzu river basin, the risk of tetR genes selection appeared to be high (5 months), moderate (5 months), and low (1 month) (Figure 5B). Patterns in Tsengwen and Yenshui river basin were similar, showing moderate (4 months) and high (1 month) risks (Figures 5C,D). Three out of four *RQ* estimates in Agongdian river basin were higher than 1, implying that OTC residues were likely to pose a high risk of tetR genes selection (Figure 5E). All the *RQ*s showed low and moderate risks, respectively, for the river sections within Kaoping and Tungkang river basins (Figures 5F,G).

**FIGURE 6:**
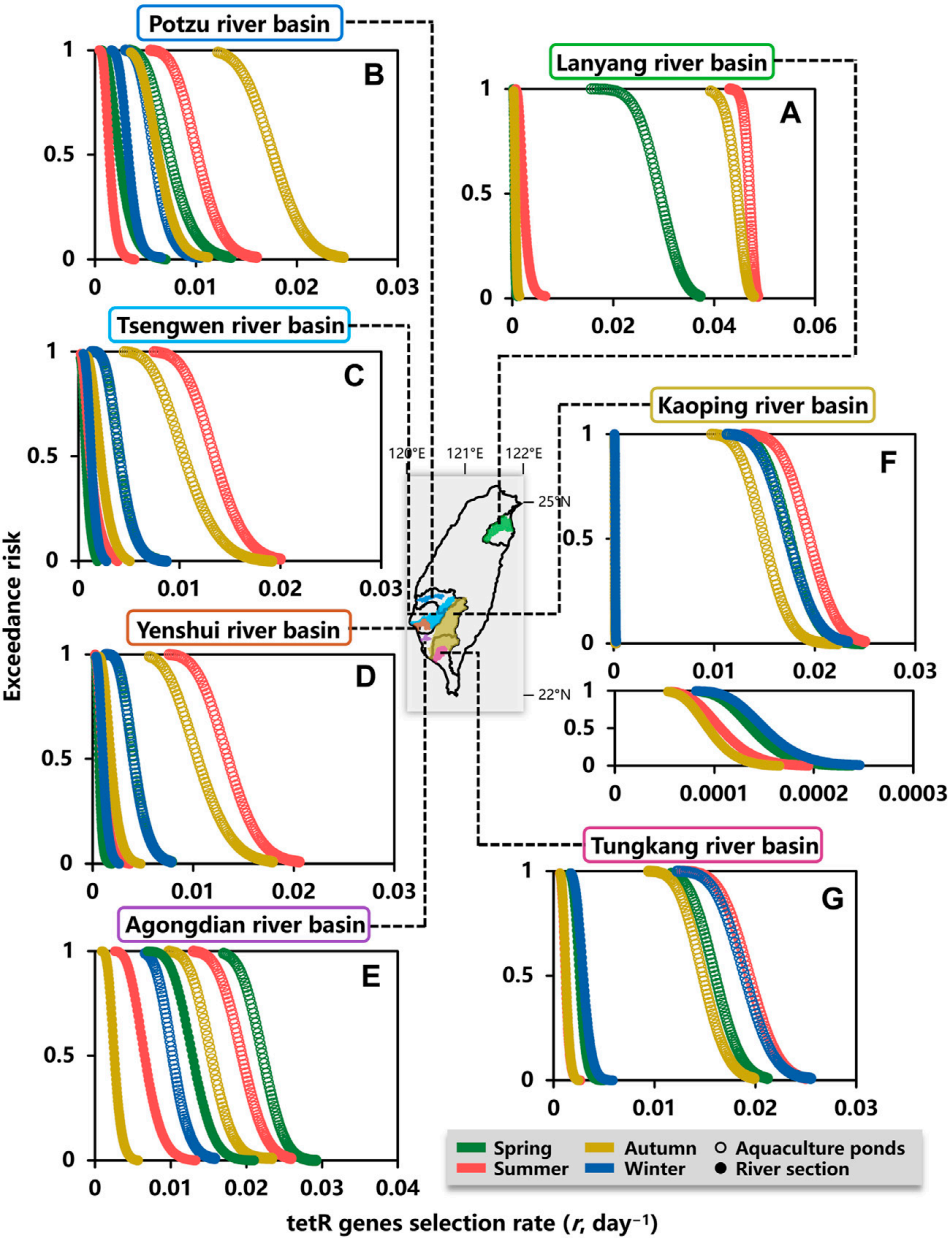
Exceedance risk (*ER*) estimates for tetracycline genes selection rate (day^−1^) in water environment of aquaculture ponds and a river section within **(A)** Lanyang, **(B)** Potzu, **(C)** Tsengwen, **(D)** Yenshui, **(E)** Agongdian, **(F)** Kaoping, and **(G)** Tungkang river basins during spring, summer, autumn, and winter.

Moreover, *ER* estimates for tetR genes selection rate (day^−1^) in water environment during spring, summer, autumn, and winter are shown in Figure 6. The tetR genes selection rates estimated at exceedance probabilities of 0.8 (*ER* = 0.8, most likely), 0.5 (*ER* = 0.5, likely), and 0.2 (*ER* = 0.2, less likely) are summarized in Supplementary Table 12. At the exceedance probability of 0.5, the highest tetR genes selection rates occurred in summer for aquaculture ponds within Lanyang, Tsengwen, Yenshui, Kaoping, and Tungkang river basins (Figures 6A,C,D,F,G), whereas the highest tetR genes selection rates for aquaculture ponds within Potzu and Agongdian river basins were in autumn and spring, respectively (Figures 6B,E).

For the river sections within Layang, Potzu, and Agongdian river basins, the seasons with the highest tetR genes selection rates were the same as those of aquaculture ponds in summer, autumn, and spring, respectively (Figures 6A,B,E). In Tsengwen, Yenshui, Kaoping, and Tungkang river basins, seasons with the highest tetR genes selection rates in the river sections were different from those of aquaculture ponds in which the highest selection rates occurred in summer (Figures 6C,D,F,G). The highest tetR genes selection rates in the river sections of Tsengwen and Yenshui river basins occurred in autumn (Figures 6C,D), whereas the highest tetR genes selection rates of Kaoping River and Tungkang River occurred in winter (Figures 6F,G).

### Control Measure Effects

A logistic function expressed as a Hill-type model could be finally developed as a control measure model to assess the effect of reduction in OTC emission rate on selection of antibiotic resistance in environments:

$$r_{\text{ARG}}\left(C_{\text{W}},P_{\text{Red}}\right)=\frac{r_{\text{ARG},\text{max}}C_{\text{W}}\left(1-P_{\text{Red}}\right)}{EC50_{\text{ARG}}+C_{\text{W}}\left(1-P_{\text{Red}}\right)},$$ (7)

where *r*_ARG_ is the tetR genes selection rate (day^−1^) as a function of specific environmental OTC concentration (*C*_W_, μg L^−1^) and the percent reduction in emission rate of OTC used in aquaculture (*P*_Red_, %). The *r*_ARG,max_ and *EC*50_ARG_ are the estimates of the Hill-1 model describing the relationship between *C*_A_ and *r* (Supplementary Table 10), defined as the maximum tetR genes selection rate (day^−1^) and concentration causing 50% of maximum tetR genes selection rate (μg L^−1^), respectively.

Based on the control model in Eq. 7, *r*_ARG_ can be simulated at a specific *C*_W_ which is reduced by *P*_Red_ and can be compared with the criteria (*r*_criterion_). The *r*_criterion_ can be *E*50 and *BMR*10 which are 50 and 10% increase in tetR selection rates, respectively, corresponding to *EC*50 and *BMC*10 in the concentration–response relationship. Therefore, minimum *P*_Red_ which enables making the tetR genes selection rate lower than *r*_criterion_ (*P*_Red,min_) could be calculated as

$$P_{\text{Red},\text{min}}=1-\frac{r_{\text{criterion}}EC50_{\text{ARG}}}{C_{\text{W}}\left(r_{\text{ARG},\text{max}}-r_{\text{criterion}}\right)}.$$ (8)

**FIGURE 7:**
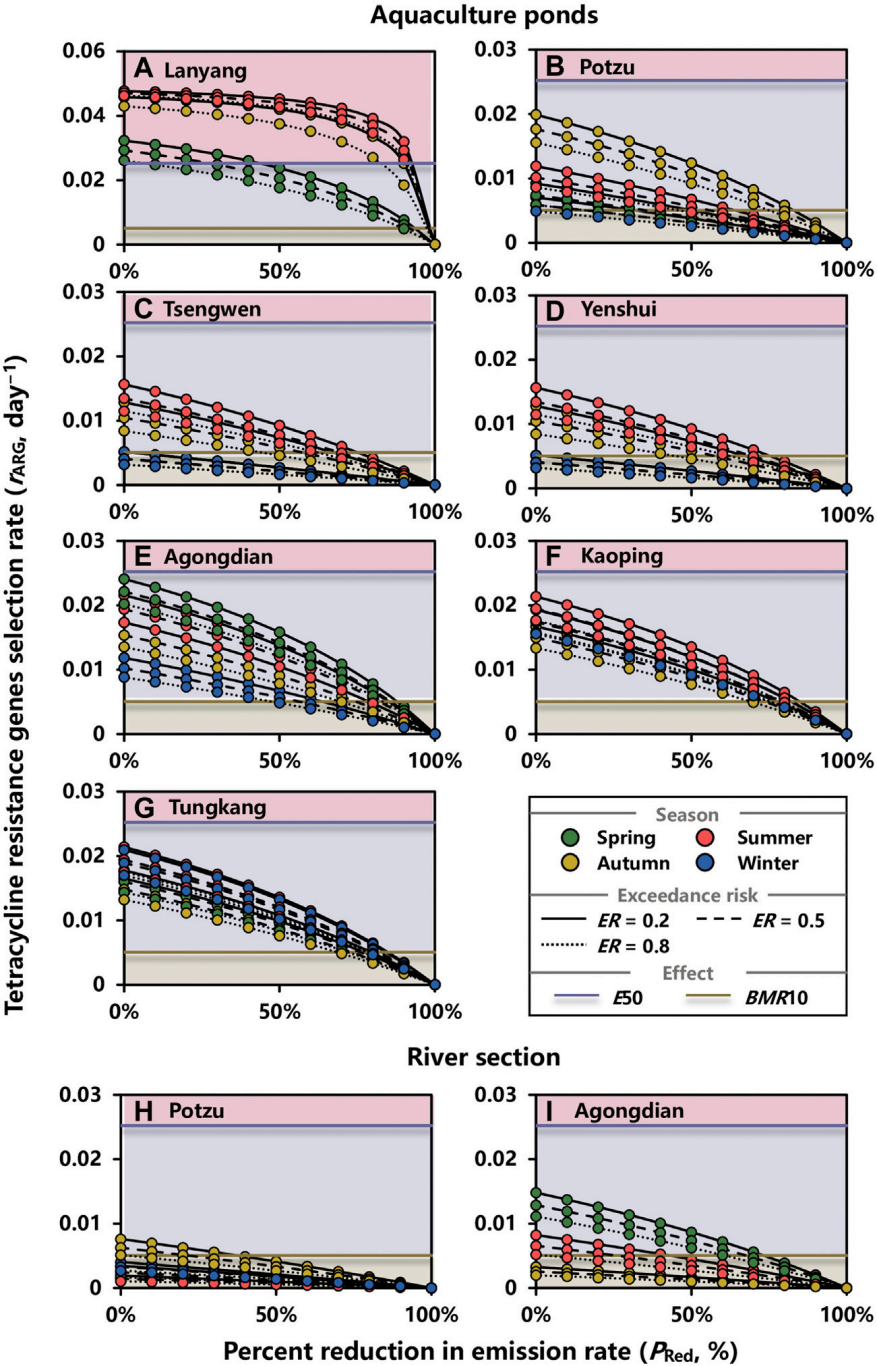
Effect of reduction in OTC emission rate on tetracycline genes selection rate (day^−1^) in water environment of **(A–G)** aquaculture ponds and **(H,I)** a river section within basins at exceedance risk (*ER*) = 0.2, 0.5, and 0.8 during spring, summer, autumn, and winter. *E*50 and *BMR*10 are 50 and 10% increase in tetracycline genes selection rate, respectively, which are corresponding to *EC*50 and *BMC*10 in the concentration–response relationship.

The effect of reduction in OTC emission on the tetR genes selection rate in aquaculture ponds at *ER* = 0.2, 0.5, and 0.8 during spring, summer, autumn, and winter are shown in Figures 7A–G. Notably, it is not easily to control the tetR genes selection rates in aquaculture ponds situated in Lanyang river basin lower than *BMR*10 in that *P*_Red,min_s were 92, 99, and 99% at *ER* = 0.5 for spring, summer, and autumn, respectively (Figure 7A; Supplementary Table 13). Moreover, if *E*50 were adopted as the criterion, reducing emission rates by 26% during spring would be fair enough; however, more efforts were needed during autumn and summer with *P*_Red,min_s of 93 and 90%, respectively (Figure 7A; Supplementary Table 13).

For other aquaculture ponds, the *P*_Red,min_s that enabled making the selection rate lower than *BMR*10 during summer were the highest in Tsengwen (69%), Yenshui (69%), Kaoping (82%), and Tungkang (82%) river basins at *ER* = 0.5, respectively (Figures 7C,D,F,G; Supplementary Table 13). For Potzu and Agongdian river basins, the highest *P*_Red,min_s were 79% during autumn and 86% during spring (Figures 7B,E; Supplementary Table 13). Although the highest *P*_Red,min_ was not shown in winter, it was unneglectable for Agongdian (56%), Kaoping (79%), and Tungkang (81%) river basins at *ER* = 0.5 (Figures 7E–G; Supplementary Table 13).

For river sections, the effects of reduction in OTC emission on the tetR genes selection rate were evaluated in Potzu and Agongdian river basins where high risk of tetR genes selection were estimated (*RQ*s > 1) (Figures 7H,I). At *ER* = 0.5, *P*_Red,min_ in the river section within Potzu river basin during autumn was 21% (Figure 7H; Supplementary Table 14). For Agongdian river basin, the concentrations of OTC during spring and summer should be given a priority to control by reducing the OTC emission rate with *P*_Red,min_s of 67 and 25%, respectively (Figure 7I; Supplementary Table 14).

**FIGURE 8:**
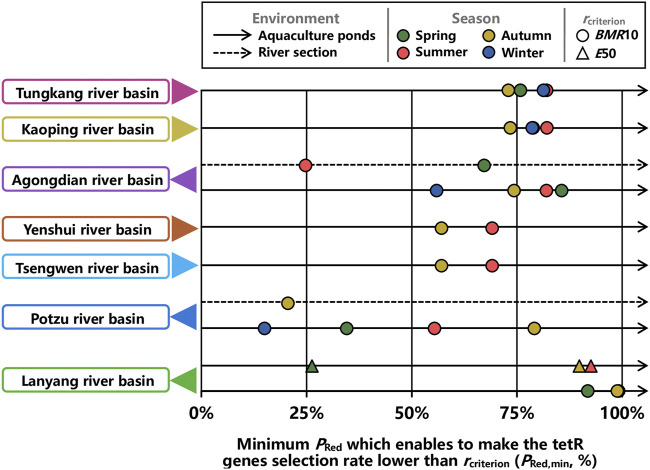
Minimum percent reduction in emission rate of OTC (*P*_Red,min_) which enables to make the tetR genes selection rate lower than a criterion (*r*_criterion_) in aquaculture ponds and river sections of seven river basins at *ER* = 0.5 during spring, summer, autumn, and winter.

To provide friendly visualizations for designing control strategies, *P*_Red,min_s at *ER* = 0.5 were summarily illustrated in Figure 8. Overall, while *BMR*10 was used as the criterion, except for Potzu river basin, *P*_Red,min_s in aquaculture ponds were higher than 50% for all seasons (Figure 8). *P*_Red,min_s in aquaculture ponds within Potzu river basin during spring and winter were lower than 50% (Figure 8). In terms of river sections, only *P*_Red,min_ in Agongdian river basin during spring was higher than 50%, whereas *P*_Red,min_s in Agongdian river basin during summer and in Potzu river basin during autumn were lower than 25% (Figure 8).

## Discussion

OTC concentrations estimated in this study fell within the range of ng L^−1^–μg L^−1^ measured in aquatic environments covering surface water, groundwater, runoff, and WWTP influent and effluent across different countries (Rico et al., 2014; Pereira et al., 2015; Xiong et al., 2015; Kim et al., 2017; Muñoz et al., 2017; Bojarski et al., 2020; Han et al., 2020). However, it should be noted that concentrations of OTC in aquaculture ponds and a river section within river basins were estimated by applying the fugacity model in which emission rates were calculated based on the annual sales of veterinary antibiotics, fisheries production data, and confirmed cases of infectious diseases instead of administration scenarios. Although the Taiwan Veterinary Drug Control Act has recommended dosage, withdrawal period, and administration route, to estimate more accurate environmental concentration of OTC, the amount of OTC used in aquaculture have to be available. Moreover, despite the indication that OTC could be used in dog, cat, chicken, pig, cow, as well as aquaculture (Lin and Tsai, 2009), in Taiwan, regulation on OTC usage could only be found in aquatic animals in Guidelines for the Use of Drugs in Animals. As a result, estimated amount of OTC among veterinary antibiotics was assumed to be totally applied in aquaculture, indicating that the present concentrations of OTC might be overestimated.

Actually, estimation of national use of antibiotic in aquaculture is complicated and varied by numerous factors such as diversity of species and culture systems, unconsolidated nature of production in many regions, and unregulated use (Henriksson et al., 2018). The likely factors influencing the application rates of antibiotics are identified as vulnerability to bacterial diseases, antibiotic assess, disease diagnostic capacity, antibiotic resistance, target markets and food safety regulations, and certification (Henriksson et al., 2018). It can be inferred that emissions of OTC from aquaculture estimated in this study are reasonable, since the factors (i.e., fisheries production of species that are approved to be treated with OTC and cases of infectious disease) included in the process of estimation are associated with application rates. However, the accuracy of emission estimates can be further improved by identifying the critical factors that affect the application rates in aquaculture situated at different regions across Taiwan.

Furthermore, studies reporting the occurrence of antibiotics in aquaculture area are limited, even though residual antibiotics not only can remain in aquaculture water and sediment but also can be released into surrounding aquatic environments, posing environmental and human health risks (Lai et al., 2018; Leal et al., 2019). Due to the data gap, we were unable to conduct model calibration and validation. As a result, we compared with the published data of OTC concentrations that were reported in two studies and a series of reports dedicated to detect emerging contaminants in aquaculture farms and rivers, respectively. In an aquaculture farm within Tsengwen river basin, the detected concentration of OTC in July was 0.08 μg L^−1^ (Lai et al., 2018), which was lower than that estimated in this study with a median concentration of 6.13 μg L^−1^ (95% CI: 3.35–11.39). The other study presented that median concentration of OTC was 12 μg L^−1^ in pond water in January 2008; however, the authors did not indicate where the aquaculture farm was situated (Lin et al., 2008).

In the series of reports of Establishing Analytical Methods for Pharmaceuticals in the Aquatic Environments (Lin and Lin, 2008, 2009, 2010), concentrations of OTC were detected in river water. OTC concentrations detected in Potzu River and Yenshui River, respectively, in September and July were both in the range of 0.01–0.02 μg L^−1^ which were lower than those estimated to be 2.57 μg L^−1^ (0.73–7.61) and 0.27 μg L^−1^ (0.09–0.72) in this study. However, OTC concentrations detected in Kaoping and Tungkang rivers, respectively, in September and July were in the range of 0.02–0.04 and 0.01–0.34 μg L^−1^ which were totally and partially covered by the estimates of 0.05 (0.02–0.10) and 0.60 μg L^−1^ (0.24–1.40), respectively. Indeed, among above detected concentrations in aquaculture farms and rivers, except for those in Kaoping and Tungkang rivers, other values were only given as a point value. Therefore, it is strongly implied that the constructed fugacity model has the capacity to reliably estimate OTC concentrations since the estimated values were within the detected values in Kaoping and Tungkang rivers.

The maximum OTC concentrations estimated in aquaculture ponds and rivers were 497.93 ± 193.03 μg L^−1^ in Lanyang river basin and 10.10 ± 3.19 μg L^−1^ in Agongdian river basin, respectively. Notably, the highest concentrations in aquaculture ponds and rivers were not presented in the same basin. This finding may be partly ascribed to difference of water flow which was proposed to be the main process responsible for occurrence of OTC in the aquatic ecosystems near fish farms (Lalumera et al., 2004). The highest estimated OTC emission rate for aquaculture ponds within Lanyang river basin was 1.8 times large than that within Agongdian river basin, whereas the discharge flow rate of aquaculture ponds within Agongdian river basin was estimated to be 12.4 times as high as that within Lanyang river basin. Higher emission rate and lower discharge flow rate of aquaculture ponds could lead to concentrations of OTC in pond water much higher than those in river water for Lanyang river basin. On the other hand, the volume of river water in Agongdian river basin was the lowest among these seven basins, partially resulting in the higher concentration of OTC in river water.

There are studies exploring the effect of aquaculture used OTC on their surrounding environment. Rico et al. (2014) conducted farm- and river-scale monitoring for OTC in Thailand. The detection rate of OTC in rivers during the wet season was higher than that during the dry season with a maximum measured concentration of 3.1 μg L^−1^. Water samples were collected from tilapia farms, environment next to the tilapia cages, and at 30 and 60 m downstream from the cages to detect OTC concentrations. Maximum OTC concentrations of 15-min post-administration were 49 and 11 μg L^−1^ inside and next to the cages, respectively. Rose and Pedersen (2009) used a Water-Quality Analysis Simulation Program model to examine the fate of OTC in streams receiving discharge from fish hatcheries. They predicted the concentrations of OTC for 8 years based on the administration scenario of 199 g day^−1^ for 12 days, in one application per year. They showed that estimated median water-column concentrations of OTC in the settling pond, receiving segment, first downstream segment, and second downstream segment were 0, 0.57, 0.80, and 0.83 ng L^−1^, respectively. It was also found that concentration could immediately decline 20- to 400-fold within 1 day of dosing.

Even though the fugacity models have been shown to successfully predict environmental concentrations of antibiotics and have been linked to performed risk assessment of antibiotics, they have not been widely applied to assess the risk of antibiotic resistance (Zhang et al., 2015; Chen et al., 2018; Dong et al., 2019). In this study, the risk of antibiotic resistance genes selection was estimated by incorporating the fugacity model into the risk assessment framework, enabling the guidance of governments to develop the risk-based management measures to reduce the amount of antibiotic entering the environment (European Medicines Agency, 2021). Since the fugacity model comprised the amount of OTC used in aquaculture, environmental behavior of OTC, and the characteristics of aquaculture farms and a river section within different river basins, results of concentration estimation were useful to inform relatively higher risk by region or point/period of time. Specific actions can be conducted in the environments considered to be at high risk of antibiotic resistance (Polianciuc et al., 2020). Moreover, monitoring of the quantities and usage patterns of antibiotics is essential for conducting risk analyses and for mitigation planning purposes. It will assist in the ability to respond to problems of antibiotic resistance in a precise and targeted way (OIE World Organisation for Animal Health, 2015). In this study, however, emission rate of OTC was assumed as a monthly-basis constant to estimate concentrations of OTC by applying the level III fugacity model. Although uncertainties from the environmental condition and emission rate were considered in the estimation, providing insightful information on the variability of the outputs of a fugacity model (Wang et al., 2020), it is suggested that the level IV fugacity model can be used to reveal the effect of unregulated use on the fate of OTC in aquaculture and river environment while the time-varying emission rate is available.

On the other hand, tetR genes, most of which are associated with mobile genetic elements (MGEs), are the largest group identified in aquaculture isolates (Piotrowska and Popowska, 2014; Shi et al., 2020). After acquiring tetR genes, microorganisms could further spread the resistance via HGT. During the process of HGT, MGEs, such as plasmids, transposons, and integrons-associated gene cassettes, play an important role in capture, accumulation, and dissemination of ARGs (van Hoek et al., 2011; Partridge et al., 2018). MGEs are capable of capturing ARGs from chromosomes and horizontally transferring them to other bacteria, promoting intracellular and intercellular DNA mobilities (Partridge et al., 2018; Ellabaan et al., 2021). However, the risk of selection of tetR genes assessed in this study was driven by OTC concentration in water only due to animal antibiotic use. In the near future, tetR that is attributed to other tetracyclines can be further studied. In addition to antibiotic residues, environmental parameters and other plausible factors such as microbial community structure and socioeconomic factors related to anthropogenic activities were found to be associated with ARG increases in river systems (Kim and Cha, 2021). Drivers of HGT have also been identified; however, their importance on HGT has not yet to be assessed at an ecological scale (Singer et al., 2016). Indeed, the characteristics of environmental media could significantly influence MGEs, bacteria community, bacterial biomass, antibiotic, and basic properties, driving the dynamics of ARGs in aquatic environments (Chen et al., 2019). Therefore, despite the fact that we could preliminarily identify the regions with relatively higher risk in specific months by considering multiple environmental media, researchers should further study the mechanism of ARG dynamics for each environmental medium specially (Chen et al., 2019).

It was also found that bacteria isolated from fish exhibited similar antibiotic resistance profiles with those of clinical isolates which have the ability to be resistant to tetracycline (Ko et al., 1996; Wu et al., 2019), suggesting that it is needed to identify the role of the environment in the transmission of antibiotic-resistant bacteria (ARB), especially those with the ability to colonize both animals and humans, to implicate intervention strategies that can aim at the sources posing risk for human exposure (Huijbers et al., 2015). To this end, it has been encouraged to prevent antibiotic overuse in aquaculture with a perspective of One Health due to the complex process of antibiotic resistance transmission at the animal–environment–human interface (Cavalli et al., 2015; Cabello et al., 2016; Lebov et al., 2017; McEwen and Collignon, 2018; Santos and Ramos, 2018; Hernando-Amado et al., 2019; Scott et al., 2019; White and Hughes, 2019). In the conceptual framework proposed for a One Health approach, minimizing the risks of antibiotic resistances is the common goal in environmental, animal, and human health and can be used as an indicator for improving control measure strategies to address antibiotic resistances problem in aquaculture.

Overall, integrating the computational fugacity model and the probabilistic risk model could estimate the risk of tetR genes selection posed by low level OTC concentrations. Results indicated that OTC residues are highly likely to pose a high risk of tetR genes selection in aquaculture ponds within all study basins, whereas risk of tetR genes selection in rivers could be variably changed in a high, moderate, and low fashion. Hence, interventions are needed to be implemented to mitigate the impact on tetR in pond water, whatever the season is and wherever aquaculture ponds situate. However, in term of rivers, where the control efforts should be focused on would be determined by discharge flow rates and volume of water, and thus the control strategies could be region-specific designed. The proposed control measure model could project the effect of reduction in OTC emission on selection of antibiotic resistance. Given a criterion of acceptable level of tetR genes selection rate, the risk-based control strategies could be provided for decision makers to zero in on environment with high risk of tetR genes selection. In the viewpoint of One Health, estimating the antibiotic concentration in the environment could be a critical step toward addressing problems of antibiotic resistance emergence and transmission due to antibiotics released from aquaculture. In conclusion, our preliminary estimates of OTC concentration in aquaculture and river basin associated with their risk estimates represent only a first step in quantifying the global importance of aquaculture used OTC-induced tetR in sustaining aquatic ecosystems. We invite further quantifications from both field measurements and more realistic descriptions of the fate of OTC flow paths in the multimedia models.

## Data Availability

The original contributions presented in the study are included in the article/Supplementary Material, and further inquiries can be directed to the corresponding author.
