# Supplementary material for: A Risk-Based Approach for Managing Aquaculture Used Oxytetracycline-Induced TetR in Surface Water Across Taiwan Regions
Source: Front Pharmacol. 2021 Dec 23;12:803499. doi: 10.3389/fphar.2021.803499 (PMC8733663; doi:10.3389/fphar.2021.803499)
Supplement: Supplementary file 1 [file DataSheet1.pdf]

SUPPLEMENTARY MATERIAL

Mathematical manipulation of environmental concentration of OTC based on the fugacity model algorithm

The fugacity capacities for water and sediment could be calculated as,

$$Z_{\text{W-Bulk}} = Z_{\text{W}}(1 - X_{\text{W-S}}) + Z_{\text{W-Solid}} X_{\text{W-S}}, \quad \text{(S1)}$$

$$Z_{\text{Sed-Bulk}} = Z_{\text{W}} X_{\text{S-W}} + Z_{\text{Sed-Solid}}(1 - X_{\text{S-W}}), \quad \text{(S2)}$$

where $Z_{\text{W-Bulk}}$ and $Z_{\text{Sed-Bulk}}$ are the bulk fugacity capacities of water and sediment (mol Pa^{-1} m^{-3}), respectively, Z_{W}, $Z_{\text{W-Solid}}$, and $Z_{\text{Sed-Solid}}$ are the fugacity capacities of water, particles in water, and particles in sediment (mol Pa^{-1} m^{-3}), respectively, $X_{\text{W-S}}$ is the volume fraction of particles in water, and $X_{\text{S-W}}$ is the volume fraction of water in sediment.

After administration, residual OTC in water could be discharged from ponds to river and then be transported via river discharge. Within aquaculture ponds and a river section, OTC could be degraded in water and sediment as well as be transported via sediment deposition, sediment resuspension, and sediment/water diffusion between water and sediment. Based on these characteristics, in the second step, transport parameters (D-values) were used to describe the transport and transformation processes in this system (Figure 2A). The mass balance equations could be established for each medium in this system as (Figure 2A),

$$E_{\text{OTC}} + (D_{\text{Res,A}} + D_{\text{Diff,A}}) f_{\text{S,A}} = (D_{\text{Dep,A}} + D_{\text{Diff,A}} + D_{\text{W-Deg,A}} + D_{\text{A}\to\text{R}}) f_{\text{W,A}}, \quad \text{(S3)}$$

$$(D_{\text{Dep,A}} + D_{\text{Diff,A}}) f_{\text{W,A}} = (D_{\text{Res,A}} + D_{\text{Diff,A}} + D_{\text{S-Deg,A}} + D_{\text{S-Bur}}) f_{\text{S,A}}, \quad \text{(S4)}$$

$$D_{\text{A}\to\text{R}} f_{\text{W,A}} + (D_{\text{Res,R}} + D_{\text{Diff,R}}) f_{\text{S,R}} = (D_{\text{Dep,R}} + D_{\text{Diff,R}} + D_{\text{W-Deg,R}} + D_{\text{Adv,R}}) f_{\text{W,R}}, \quad \text{(S5)}$$

$$(D_{\text{Dep,R}} + D_{\text{Diff,R}}) f_{\text{W,R}} = (D_{\text{Res,R}} + D_{\text{Diff,R}} + D_{\text{S-Deg,R}}) f_{\text{S,R}}, \quad \text{(S6)}$$

where E_{OTC} is the emission rate of OTC (mol h^{-1}), f_{W} and f_{S} are the fugacities for the bulk of water and sediment (Pa), respectively, subscripts A and R represent the values for aquaculture ponds and a river section, respectively, and D_{Dep}, D_{Res}, D_{Diff}, $D_{\text{W-Deg}}$, $D_{\text{S-Deg}}$, $D_{\text{S-Bur}}$, $D_{\text{A}\to\text{R}}$, and $D_{\text{Adv,R}}$ are the D-values for the processes of sediment deposition, sediment resuspension, sediment/water diffusion, degradation in water, degradation in sediment, sediment burial, discharge from aquaculture ponds, and river discharge, respectively (mol Pa^{-1} h^{-1}).

The process of estimating OTC concentrations in water and sediment by the fugacity model is illustrated in Figure 2B. To perform the mathematical manipulation, we let

$$(D_{\text{Dep,A}} + D_{\text{Diff,A}} + D_{\text{W-Deg,A}} + D_{\text{A}\to\text{R}}) = D_{\text{Tot-W,A}}, \quad \text{(S7)}$$

$$(D_{\text{Res,A}} + D_{\text{Diff,A}} + D_{\text{S-Deg,A}} + D_{\text{S-Bur}}) = D_{\text{Tot-S,A}}, \quad \text{(S8)}$$

$$(D_{\text{Dep,R}} + D_{\text{Diff,R}} + D_{\text{W-Deg,R}} + D_{\text{Adv,R}}) = D_{\text{Tot-W,R}}, \quad \text{(S9)}$$

$$(D_{\text{Res,R}} + D_{\text{Diff,R}} + D_{\text{S-Deg,R}}) = D_{\text{Tot-S,R}}. \quad \text{(S10)}$$

Solving for $f_{\text{W,A}}$, $f_{\text{S,A}}$, $f_{\text{W,R}}$, and $f_{\text{S,R}}$ gives,

$$f_{\mathrm{W,A}} = \frac{E_{\mathrm{OTC}} D_{\mathrm{Tot-S,A}}}{D_{\mathrm{Tot-W,A}} D_{\mathrm{Tot-S,A}} - (D_{\mathrm{Res,A}} + D_{\mathrm{Diff,A}})(D_{\mathrm{Dep,A}} + D_{\mathrm{Diff,A}})}, \quad \text{(S11)}$$

$$f_{\mathrm{S,A}} = \frac{E_{\mathrm{OTC}} (D_{\mathrm{Dep,A}} + D_{\mathrm{Diff,A}})}{D_{\mathrm{Tot-W,A}} D_{\mathrm{Tot-S,A}} - (D_{\mathrm{Res,A}} + D_{\mathrm{Diff,A}})(D_{\mathrm{Dep,A}} + D_{\mathrm{Diff,A}})}, \quad \text{(S12)}$$

$$f_{\mathrm{W,R}} = \frac{D_{\mathrm{A \to R}} f_{\mathrm{W,A}} D_{\mathrm{Tot-S,R}}}{D_{\mathrm{Tot-W,R}} D_{\mathrm{Tot-S,R}} - (D_{\mathrm{Res,R}} + D_{\mathrm{Diff,R}})(D_{\mathrm{Dep,R}} + D_{\mathrm{Diff,R}})}, \quad \text{(S13)}$$

$$f_{\mathrm{S,R}} = \frac{D_{\mathrm{A \to R}} f_{\mathrm{W,A}} (D_{\mathrm{Dep,R}} + D_{\mathrm{Diff,R}})}{D_{\mathrm{Tot-W,R}} D_{\mathrm{Tot-S,R}} - (D_{\mathrm{Res,R}} + D_{\mathrm{Diff,R}})(D_{\mathrm{Dep,R}} + D_{\mathrm{Diff,R}})}. \quad \text{(S14)}$$

Therefore, environmental concentrations of OTC in bulks of water (C_{W}, mol m^{-3}) and sediment (C_{S}, mol m^{-3}) of aquaculture ponds and a river section could be obtained by multiplying the fugacity (*f*) and the fugacity capacity (*Z*) as

$$C_{\mathrm{W,A}} = Z_{\mathrm{W-Bulk}} f_{\mathrm{W,A}}, \quad \text{(S15)}$$

$$C_{\mathrm{S,A}} = Z_{\mathrm{S-Bulk}} f_{\mathrm{S,A}}, \quad \text{(S16)}$$

$$C_{\mathrm{W,R}} = Z_{\mathrm{W-Bulk}} f_{\mathrm{W,R}}, \quad \text{(S17)}$$

$$C_{\mathrm{S,R}} = Z_{\mathrm{S-Bulk}} f_{\mathrm{S,R}}. \quad \text{(S18)}$$

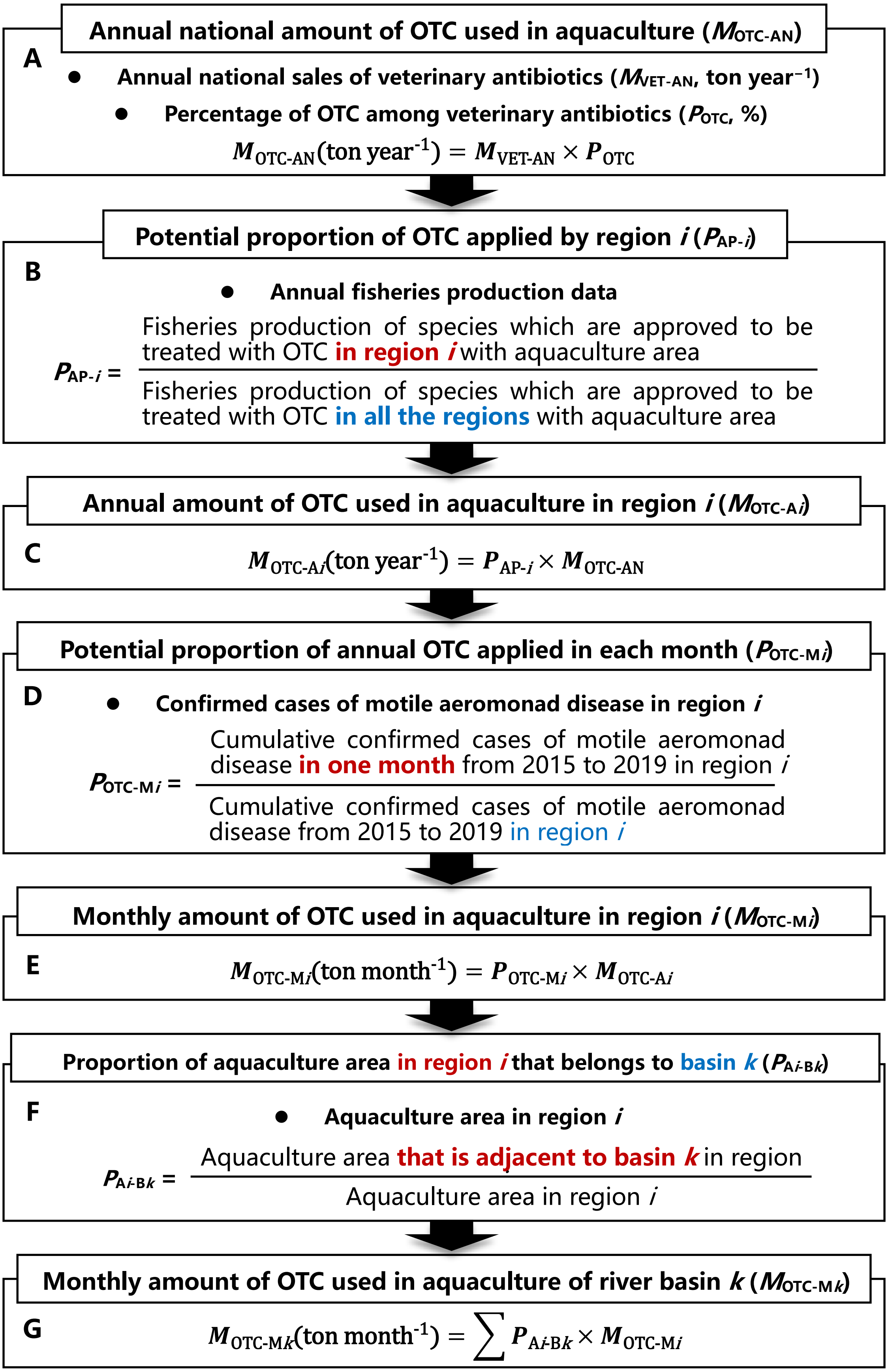

Supplementary Figure 1. Flowchart of estimating monthly amount of OTC used in aquaculture in river basin.

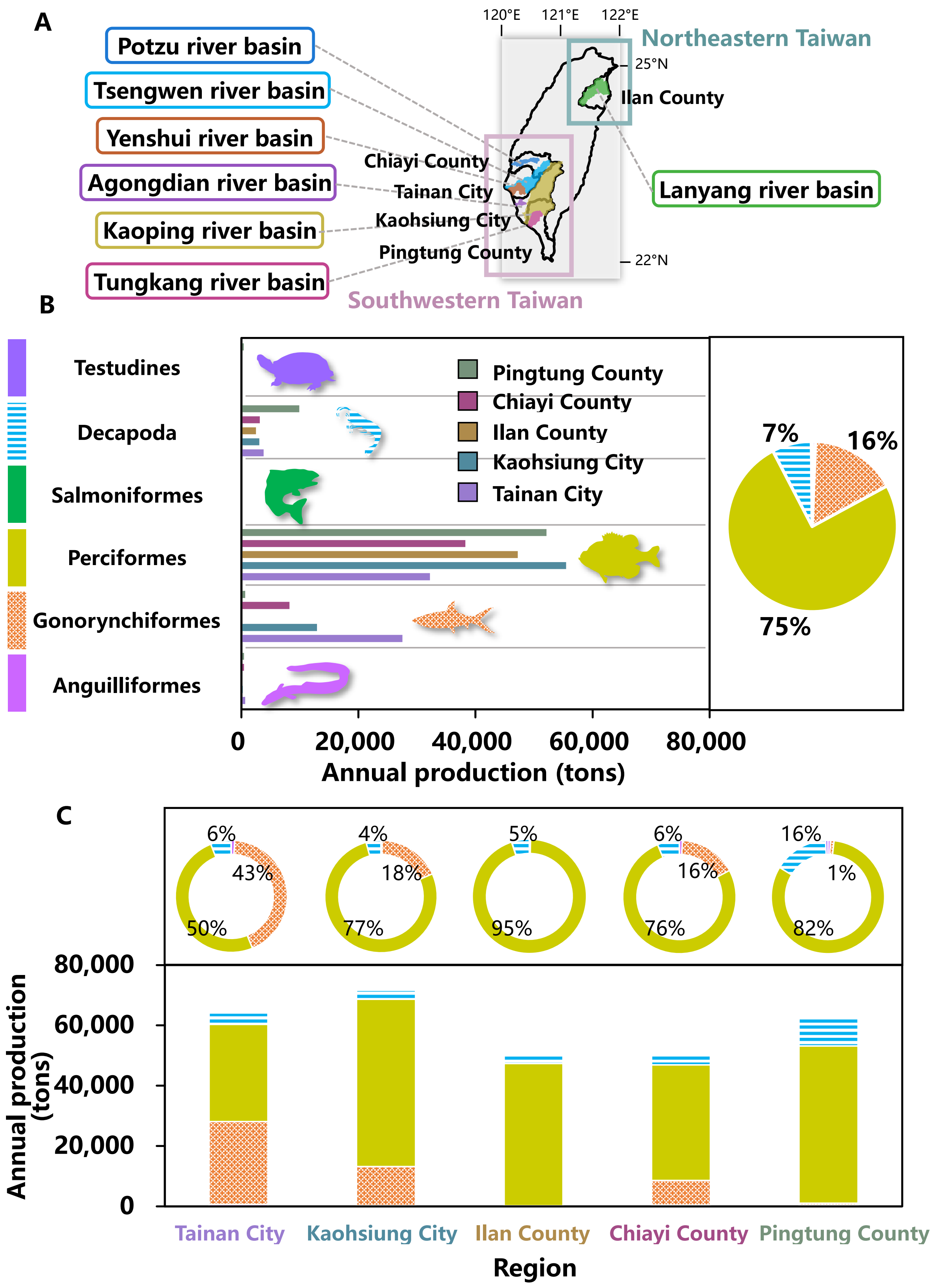

Supplementary Figure 2. (**A**) Seven study river basins situated at southwestern and northeastern areas selected based on the regions with (**B**) top 5 annual average fisheries production and (**C**) percentage of species that are approved to be treated with OTC, including seven orders of Anguilliformes, Gonorynchiformes, Perciformes, Salmoniformes, Decapoda, Anura, and Testudines.

Supplementary Table 1. Parameters for estimating monthly amount of OTC used in aquaculture in a basin

**Parameter**	**Value for region *i***				
	Kaohsiung City	Tainan City	Pingtung County	Chiayi County	Ilan County
$M_{\text{VET-AN}}$ [a]	N(1276800, 192820) [b]				
P_{OTC} [c]	N(6.86, 1.83)				
$P_{\text{AP-}i}$ [d]	N(0.201, 0.008)	N(0.180, 0.010)	N(0.178, 0.004)	N(0.141, 0.020)	N(0.140, 0.019)
$P_{\text{OTC-M}i}$ [e]					
January	0	0	0.046	0.023	0
February	0.045	0	0.092	0.070	0
March	0.090	0	0.072	0	0.030
April	0.134	0	0.078	0.023	0.030
May	0.150	0.111	0.072	0.140	0
June	0.180	0.333	0.059	0.093	0.152
July	0.030	0.111	0.111	0.023	0.152
August	0.090	0	0.131	0.116	0.303
September	0.134	0	0.072	0.233	0.273
October	0.030	0.333	0.059	0.116	0.061
November	0.045	0	0.065	0.140	0
December	0.075	0.111	0.144	0.023	0
$P_{\text{A}i\text{-B}k}$ [f]					
Lanyang river basin					100
Potzu river basin				100	
Tsengwen river basin		54.14			
Yenshui river basin		21.52			
Kaoping river basin	7.78		32.84		
Agongdian river basin	62.53				
Tungkang river basin			32.28		

[a] Estimated based on the total sales of veterinary antibiotics in Taiwan from 2015 to 2019 (https://www.agriharvest.tw/archives/45733).

[b] N($\bar{x}$, SD) denotes the normal distribution with mean $\bar{x}$ and standard deviation SD.

[c] Estimated based on Chen et al. (2019).

[d] Estimated based on the Annual Publication of Fishery Statistics from 2015 to 2019 published by Fisheries Agency, Council of Agriculture (FACOA), Executive Yuan (FACOA, 2021).

[e] Estimated based on the confirmed cases of motile aeromonad disease from 2015 to 2019 reported by Bureau of Animal and Plant Health Inspection and Quarantine (BAPHIQ), Council of Agriculture, Executive Yuan (BAPHIQ, 2021).

[f] Estimated based on the annual publication published by the Department of Budget, Accounting and Statistics.

Supplementary Table 2. Datasets of tetR genes and transposon selection rates at different exposure concentrations of OTC extracted from Knapp et al. (2008)

OTC concentration (μg L^{-1})	Selection rate (day^{-1})	
	tetR genes	Transposon
0	0.013 ± 0.030 [a]	0.015 ± 0.040
5	0.017 ± 0.022	0.013 ± 0.034
20	0.020 ± 0.010	0.064 ± 0.031
50	0.030 ± 0.016	0.050 ± 0.028
250	0.049 ± 0.016	0.092 ± 0.032

[a] Mean ± SD.

Supplementary Table 3. Equations of Z-values (mol Pa^{-1} m^{-3}) for environmental media

**Medium**	**Z-value**	**Equation**
Water	Z_{W}	$1/H$
Particles in water	$Z_{W\text{-}Solid}$	$m_{OC(W)}K_{OC}Z_{W}\rho_{S(W)}/1000$
Particles in sediment	$Z_{Sed\text{-}Solid}$	$m_{OC(Sed)}K_{OC}Z_{W}\rho_{S(Sed)}/1000$
Bulk of water	$Z_{W\text{-}Bulk}$	$Z_{W}(1 - X_{W\text{-}Solid}) + Z_{W\text{-}Solid}(X_{W\text{-}Solid})$
Bulk of sediment	$Z_{Sed\text{-}Bulk}$	$Z_{W}(X_{Sed\text{-}W}) + Z_{Sed\text{-}Solid}\ (1 - X_{Sed\text{-}W})$

Supplementary Table 4. General parameters of fugacity model for calculating Z-values

Parameter	Value	Unit	Reference
Henry's law constant (H)	3.96×10^{-21}	Pa m^3 mol^{-1}	Leal et al. (2019)
Mass fraction organic carbon in water ($m_{OC(W)}$)	0.018	Dimensionless	Steeby et al. (2004)
Mass fraction organic carbon in sediment ($m_{OC(Sed)}$)	0.018	Dimensionless	Steeby et al. (2004)
Organic carbon–water partition ratio (K_{OC})	1.4×10^{3}	L/kg	Chen et al. (2018)
Density of solid in water of aquaculture ponds ($\rho_{S(W\text{-}A)}$)	475	kg m^{-3}	Boyd et al. (2010)
Density of solid in sediment of aquaculture ponds ($\rho_{S(Sed\text{-}A)}$)	475	kg m^{-3}	Boyd et al. (2010)
Density of solid in water of river ($\rho_{S(W\text{-}R)}$)	2400	kg m^{-3}	Zhang et al. (2015)
Density of solid in sediment of river ($\rho_{S(Sed\text{-}R)}$)	2400	kg m^{-3}	Zhang et al. (2015)
Volume fraction of particles in water ($X_{W\text{-}Solid}$)	1.3×10^{-3}	Dimensionless	Chen et al. (2018)
Volume fraction of water in sediment ($X_{Sed\text{-}W}$)	0.7	Dimensionless	Chen et al. (2018)

Supplementary Table 5. Equations of D-values (mol Pa^{-1} h^{-1}) for transport processes

**Process**	**D-value**	**Equation**
Degradation in water	$D_{W\text{-}Deg}$	$k_{WR}V_W Z_W$
Water outflow	D_{Out}	$G_{Out}Z_W$
Discharge from aquaculture ponds	$D_{A\rightarrow R}$	$G_{A\rightarrow R}Z_W$
River discharge	$D_{Adv,R}$	$G_{Adv}Z_W$
Sediment deposition	D_{Dep}	$A_{W\text{-}S}K_{Dep}Z_{W\text{-}Solid}$
Sediment resuspension	D_{Res}	$A_{W\text{-}S}K_{Res}Z_{Sed\text{-}Solid}$
Sediment/Water diffusion	D_{Diff}	$1/(1/k_{W\text{-}S}A_{W\text{-}S}Z_W + Y_S/B_S A_{W\text{-}S}Z_W)$
Degradation in sediment	$D_{S\text{-}Deg}$	$k_{SR}V_S Z_{Sed\text{-}Bulk}$
Burial	$D_{S\text{-}Bur}$	$G_{Bur}Z_{Sed\text{-}Solid}$

Supplementary Table 6. General parameters of fugacity model for calculating D-values

Parameter	Value	Unit	Reference
**Parameter for aquaculture ponds and a river section**			
Degradation rate in water (k_{WR})	$0.693/(2.16\times10^{2})$	h^{-1}	Zhang et al. (2015)
Degradation rate in sediment (k_{SR})	$0.693/(1.75\times10^{3})$	h^{-1}	Zhang et al. (2015)
Water-side mass transfer coefficient over sediment ($k_{W\text{-}S}$)	0.01	$m\ h^{-1}$	Chen et al. (2018)
Diffusion path lengths in sediment (Y_S)	0.3	m	Jiménez-Montealegre et al. (2002)
Molecular diffusivity in sediment (B_S)	8.30×10^{-8}	$m^2\ h^{-1}$	Zhang et al. (2015)
Sediment deposition rate (K_{Dep})	3.9×10^{-5}	$m\ h^{-1}$	Zhang et al. (2015)
Sediment resuspension rate (K_{Res})	1.14×10^{-8}	$m\ h^{-1}$	Zhang et al. (2015)
Volume of water (V_W)	$A_{W\text{-}S}\times d_W$	m^3	
Volume of sediment (V_S)	$A_{W\text{-}S}\times d_S$	m^3	
**Parameter for aquaculture ponds**			
Depth of pond water (d_W)	1	m	Baluyut (1989)
Depth of sediment ($d_{S\text{-}A}$)	0.17	m	Boyd et al. (2010)
Burial rate (G_{Bur})	$L_{Burial}A_{W\text{-}S,A}/(\rho_{S(Sed\text{-}A)}\times365\times24\times10^{6})$	$m^3\ h^{-1}$	
Burial flux (L_{Burial})	148.9	$g\ m^{-2}\ yr^{-1}$	Boyd et al. (2010)
**Parameter for a river section**			
Depth of sediment ($d_{S\text{-}R}$)	0.05	m	Zhang et al. (2015)

Supplementary Table 7. Basin-specific parameters of fugacity model for aquaculture ponds and a river section

Parameter	Mon.	Lanyang	Potzu	Tsengwen	Yenshui	Agongdian	Kaoping	Tungkang
**Aquaculture ponds**								
Area of water phase ($A_{W\text{-}S,A}$, m^2) [a]		$2.12\times10^6 \pm 4.22\times10^5$ [b]	$4.34\times10^7 \pm 4.04\times10^6$	$4.17\times10^7 \pm 2.06\times10^6$	$1.66\times10^7 \pm 8.19\times10^5$	$2.02\times10^7 \pm 4.68\times10^5$	$1.09\times10^7 \pm 5.36\times10^5$	$8.26\times10^6 \pm 5.23\times10^5$
Discharge flow rate ($G_{A\rightarrow R}$, m^3 h^{-1}) [c]		577.97	15231.53	7022.62	2790.87	7173.53	7766.38	6756.75
**River section**								
River width (W_R, m) [d]		266.12	165.52	236.48	123.42	110.69	466.83	133.72
Area of water phase ($A_{W\text{-}S,R}$, m^2) [e]		1.65×10^6	3.52×10^6	6.28×10^6	1.57×10^6	8.34×10^5	1.57×10^7	2.28×10^6
Volume of water (V_W, km^3) [f]	Jan	4.850 ± 0.7 [f]	2.22 ± 0.82	1.25 ± 0.46	0.60 ± 0.09	0.54 ± 0.20	374.05 ± 4.95	10.10 ± 0.33
	Feb	4.686 ± 0.5	2.14 ± 0.68	1.31 ± 0.30	0.62 ± 0.08	0.53 ± 0.20	373.21 ± 4.65	9.99 ± 0.32
	Mar	4.636 ± 0.4	2.17 ± 0.45	1.70 ± 0.31	0.69 ± 0.13	0.53 ± 0.19	373.11 ± 5.16	9.91 ± 0.30
	Apr	4.525 ± 0.4	2.42 ± 0.63	2.11 ± 0.69	0.75 ± 0.08	0.49 ± 0.14	373.84 ± 7.22	9.82 ± 0.33
	May	4.697 ± 0.6	2.78 ± 1.02	2.48 ± 0.60	0.81 ± 0.09	0.54 ± 0.09	378.31 ± 2.36	10.00 ± 0.34
	Jun	4.920 ± 0.7	4.18 ± 0.59	3.88 ± 1.28	0.96 ± 0.11	0.65 ± 0.15	385.10 ± 8.00	11.15 ± 0.64
	Jul	4.753 ± 0.5	4.32 ± 0.56	4.36 ± 0.60	1.15 ± 0.09	0.71 ± 0.13	384.80 ± 3.97	11.77 ± 0.63
	Aug	5.099 ± 0.4	5.18 ± 1.39	6.07 ± 2.36	1.29 ± 0.21	0.87 ± 0.22	388.41 ± 2.99	12.39 ± 0.83
	Sep	5.497 ± 0.2	4.18 ± 1.27	4.82 ± 2.42	1.16 ± 0.23	0.69 ± 0.22	385.03 ± 9.57	11.90 ± 1.15
	Oct	5.876 ± 0.5	2.90 ± 0.93	2.71 ± 0.25	1.05 ± 0.27	0.52 ± 0.17	379.45 ± 9.39	10.93 ± 0.62
	Nov	5.492 ± 0.4	2.29 ± 0.61	2.01 ± 0.17	0.83 ± 0.13	0.51 ± 0.21	373.29 ± 7.50	10.41 ± 0.38
	Dec	5.313 ± 0.3	2.00 ± 0.66	1.44 ± 0.18	0.66 ± 0.05	0.50 ± 0.25	370.84 ± 7.19	10.22 ± 0.33
Discharge flow rate (G_{Adv}, m^3 sec^{-1}) [g]	Jan	42.81 ± 23.17	3.86 ± 0.10	1.21 ± 1.22	1.31 ± 0.78		48.99 ± 40.81	8.33 ± 4.22
	Feb	30.68 ± 12.06	3.80 ± 0.63	1.12 ± 1.02	2.13 ± 0.98		43.17 ± 42.91	6.66 ± 3.47
	Mar	26.59 ± 8.74	5.52 ± 1.87	1.16 ± 1.19	1.83 ± 0.79	3.09 [h]	48.38 ± 42.84	5.61 ± 2.79
	Apr	21.39 ± 6.90	7.42 ± 4.09	1.97 ± 2.94	3.11 ± 2.67		65.17 ± 58.61	5.52 ± 2.85
	May	35.64 ± 20.36	12.48 ± 8.03	7.58 ± 10.18	3.70 ± 1.21	3.35	147.76 ± 138.17	8.02 ± 2.43

Jun	57.69 ± 44.42	28.10 ± 14.70	36.88 ± 54.88	13.64 ± 9.74		335.41 ± 300.27	27.82 ± 14.86
Jul	37.80 ± 16.08	28.32 ± 11.46	33.50 ± 15.61	17.70 ± 9.04		280.16 ± 184.31	41.00 ± 19.00
Aug	68.07 ± 55.30	49.51 ± 31.29	127.39 ± 127.70	25.46 ± 23.59	5.89	417.09 ± 319.19	53.84 ± 26.54
Sep	92.25 ± 5.98	27.53 ± 18.72	52.82 ± 56.18	16.16 ± 20.60		339.62 ± 393.27	43.35 ± 27.32
Oct	225.46 ± 225.89	10.04 ± 2.87	5.58 ± 4.45	3.68 ± 1.56	5.7	154.50 ± 133.99	23.36 ± 12.66
Nov	87.96 ± 18.37	5.85 ± 1.14	0.94 ± 0.54	2.47 ± 1.46		52.04 ± 34.20	13.41 ± 6.84
Dec	67.48 ± 29.48	4.19 ± 0.50	1.61 ± 2.02	1.88 ± 1.15		34.74 ± 23.83	9.95 ± 5.25

[a] Estimated based on the data adopted from Annual Publication of Fishery Statistics published by Fisheries Agency, Council of Agriculture, Executive Yuan.

[b] Mean ± SD.

[c] Estimated based on the data adopted from Annual Water Consumption Report published by Water Resources Agency, Ministry of Economic Affairs.

[d] Estimated based on the relationship between river width (W_R, m) and catchment area (A_S, km^2) that was constructed by Hsieh et al. (2013) as, $W_R = 13.36A_S^{0.45}$. A_S for a river section in a basin was adopted from Hydrological Year Book of Taiwan Republic of China (WRA, 2015 – 2019).

[e] $A_{W\text{-}S,R}$ = Length of a river section × W_R. Length of the river sections in Lanyang, Potzu, Tsengwen, Yenshui, Kaoping, Agongdian, and Tungkang River basins were adopted from Northern Region Water Resources Office (2014), The 5th River Management Office, WRA (2005), Tsai (2006), WRA (2015b), The 7th River Management Office, WRA (2011), Water Resources Planning Institute, WRA (2012), and WRA (2011), respectively.

[f] $V_W = A_{W\text{-}S,R}$ × (Depth of water in a river section, m) × 10^{-6}. Depth of water in a river section was estimated based on monthly average level of water that was adopted from Hydrological Year Book of Taiwan Republic of China (WRA, 2015 – 2019).

[g] Estimated based on monthly average discharge flow rate that was adopted from Hydrological Year Book of Taiwan Republic of China (WRA, 2015 – 2019).

[h] Single value.

Supplementary Table 8. Estimated OTC emission rates (mol h^{-1}) for aquaculture ponds within a river basin in different months

Month	Lanyang	Potzu	Tsengwen	Yenshui	Agongdian	Kaoping	Tungkang
Jan	0	0.82 ± 0.28 [a]	0	0	0	0.67 ± 0.21	0.66 ± 0.20
Feb	0	2.72 ± 0.94	0	0	1.56 ± 0.49	1.68 ± 0.52	1.46 ± 0.45
Mar	1.06 ± 0.36	0	0	0	2.82 ± 0.88	1.40 ± 0.44	1.03 ± 0.32
Apr	1.09 ± 0.37	0.85 ± 0.29	0	0	4.37 ± 1.37	1.73 ± 0.54	1.17 ± 0.36
May	0	4.92 ± 1.70	2.71 ± 0.85	1.08 ± 0.34	4.70 ± 1.47	1.64 ± 0.51	1.03 ± 0.32
Jun	5.46 ± 1.85	3.39 ± 1.17	8.39 ± 2.64	3.34 ± 1.05	5.82 ± 1.82	1.61 ± 0.50	0.87 ± 0.27
Jul	5.28 ± 1.79	0.82 ± 0.28	2.71 ± 0.85	1.08 ± 0.34	0.94 ± 0.29	1.74 ± 0.54	1.60 ± 0.50
Aug	10.57 ± 3.59	4.10 ± 1.42	0	0	2.82 ± 0.88	2.26 ± 0.70	1.88 ± 0.58
Sep	9.83 ± 3.34	8.47 ± 2.93	0	0	4.37 ± 1.37	1.63 ± 0.51	1.07 ± 0.33
Oct	2.11 ± 0.72	4.10 ± 1.42	8.12 ± 2.56	3.23 ± 1.02	0.94 ± 0.29	0.98 ± 0.30	0.85 ± 0.26
Nov	0	5.08 ± 1.76	0	0	1.46 ± 0.46	1.17 ± 0.36	0.97 ± 0.30
Dec	0	0.82 ± 0.28	2.71 ± 0.85	1.08 ± 0.34	2.35 ± 0.73	2.40 ± 0.74	2.07 ± 0.64

[a] Mean ± SD.

Supplementary Table 9. Estimated monthly concentrations of OTC (μg L^{-1}) in water of aquaculture ponds (AP) and a river section (RS) that affected by discharges from aquaculture

Month	Env.	Lanyang	Potzu	Tsengwen	Yenshui	Agongdian	Kaoping	Tungkang
Jan	AP		1.80 ± 0.65 [a]				5.33 ± 1.66	6.81 ± 2.17
	RS		0.96 ± 0.37				0.03 ± 0.01	0.71 ± 0.27
Feb	AP		5.98 ± 2.13			7.35 ± 2.35	13.42 ± 4.26	15.04 ± 4.76
	RS		3.19 ± 1.19				0.08 ± 0.03	1.71 ± 0.64
Mar	AP	50.17 ± 19.79				13.26 ± 4.17	11.21 ± 3.52	10.67 ± 3.39
	RS	0.27 ± 0.13				6.44 ± 2.04	0.06 ± 0.02	1.29 ± 0.46
Apr	AP	51.71 ± 20.25	1.86 ± 0.67			20.52 ± 6.47	13.82 ± 4.34	11.99 ± 3.75
	RS	0.33 ± 0.16	0.73 ± 0.35				0.07 ± 0.02	1.47 ± 0.52
May	AP		10.83 ± 3.84	6.42 ± 2.07	6.46 ± 2.08	22.13 ± 6.99	12.99 ± 4.05	10.62 ± 3.40
	RS		3.14 ± 1.71	1.14 ± 0.57	0.97 ± 0.37	10.10 ± 3.19	0.06 ± 0.02	1.10 ± 0.38
Jun	AP	257.63 ± 100.79	7.41 ± 2.63	19.98 ± 6.30	19.93 ± 6.35	27.39 ± 8.63	12.85 ± 4.00	9.06 ± 2.88
	RS	0.94 ± 0.71	1.07 ± 0.58	1.59 ± 1.17	1.33 ±0.86		0.05 ± 0.02	0.49 ± 0.23
Jul	AP	249.56 ± 98.42	1.81 ± 0.65	6.47 ± 2.08	6.46 ± 2.07	4.41 ± 1.38	13.83 ± 4.35	16.53 ± 5.29
	RS	1.05 ± 0.57	0.25 ± 0.12	0.34 ± 0.17	0.30 ± 0.17		0.05 ± 0.02	0.66 ± 0.30
Aug	AP	497.93 ± 193.03	9.00 ± 3.22			13.18 ± 4.13	18.00 ± 5.69	19.25 ± 6.08
	RS	1.62 ± 1.27	0.85 ± 0.54			3.65 ± 1.15	0.06 ± 0.02	0.62 ± 0.31
Sep	AP	463.76 ± 184.38	18.62 ± 6.74			20.45 ± 6.34	13.02 ± 4.03	10.96 ± 3.41
	RS	0.76 ±0.31	2.99 ± 1.85				0.05 ±0.02	0.45 ± 0.24
Oct	AP	99.73 ± 38.42	8.97 ± 3.22	19.48 ± 6.23	19.34 ± 6.10	4.41 ± 1.40	7.86 ± 2.51	8.70 ± 2.76
	RS	0.131 ±0.129	2.64 ± 1.08	3.45 ± 1.46	2.84 ± 1.18	1.31 ± 0.42	0.04 ± 0.01	0.53 ± 0.24
Nov	AP		11.13 ± 3.99			6.83 ± 2.13	9.35 ± 2.98	10.01 ± 3.15
	RS		4.70 ± 1.79				0.05 ± 0.02	0.83 ± 0.33
Dec	AP		1.80 ± 0.65	6.52 ± 2.11	6.46 ± 2.06	10.98 ± 3.50	19.04 ± 5.82	21.31 ± 6.74
	RS		0.93 ± 0.35	1.91 ± 0.71	1.56 ± 0.66		0.11 ± 0.03	2.05 ± 0.80

[a] Mean ± SD.

Supplementary Table 10. Fitted coefficients of the Hill-1 model[a] describing the relationship between OTC concentration and selection rates of tetracycline resistance (tetR) genes and transposon (Mean and standard deviation (SD) of data adopted from Knapp et al. (2008))

Coefficient	tetR gene	Transposon
**r_{max} (day^{-1})**	0.05 ± 0.01** [b]	0.09 ± 0.02***
***EC*50 (μg L^{-1})**	23.27 ± 21.39	15.34 ± 12.74
***BMC*10 (μg L^{-1})**	2.59 (95% CI: 0.97 – 4.21)	1.71 (0.67 – 2.75)
**r^2**	0.12	0.38*

[a]The Hill-1 model was described as, $r(C_A) = \frac{r_{max}C_A}{EC50+C_A}$ where C_A is the OTC concentration in water (μg L^{-1}), $r(C_A)$ is the selection rate as a function of specific OTC concentration (day^{-1}), r_{max} is the maximum selection rate (day^{-1}), *EC*50 is the concentration causing 50% of maximum selection rate (μg L^{-1}).

[b] Mean ± SE.

* p-value < 0.05, ** p-value < 0.01, *** p-value < 0.001.

Supplementary Table 11. Monthly risk quotients (*RQ*s) for tetracycline genes selection in water environment of aquaculture ponds (AP) and a river section (RS), respectively, at 2.5, 25, 50, 75, and 97.5% probabilities subject to OTC residues

Mon	Probability	Lanyang		Potzu		Tsengwen		Yenshui		Agongdian		Kaoping		Tungkang	
		AP	RS	AP	RS	AP	RS	AP	RS	AP	RS	AP	RS	AP	RS
**Jan**	**2.5%**			0.24	0.12							0.75	0.004	0.84	0.09
	**25%**			0.50	0.26							1.51	0.008	1.92	0.19
	**50%**			0.73	0.38							2.16	0.012	2.77	0.28
	**75%**			1.06	0.56							3.13	0.017	4.01	0.42
	**97.5%**			2.17	1.19							6.29	0.035	8.18	0.89
**Feb**	**2.5%**			0.79	0.41					1.04		1.89	0.010	2.10	0.22
	**25%**			1.65	0.87					2.04		3.78	0.021	4.24	0.46
	**50%**			2.39	1.27					2.99		5.45	0.031	6.13	0.69
	**75%**			3.54	1.88					4.36		7.95	0.045	8.80	1.02
	**97.5%**			7.14	3.85					8.85		16.13	0.091	17.65	2.15
**Mar**	**2.5%**	6.34	0.03							1.88	0.91	1.55	0.008	1.48	0.17
	**25%**	13.38	0.07							3.75	1.82	3.15	0.017	3.02	0.36
	**50%**	19.74	0.10							5.44	2.64	4.59	0.025	4.31	0.52
	**75%**	29.63	0.16							7.76	3.79	6.60	0.037	6.28	0.77
	**97.5%**	64.16	0.37							15.25	7.46	13.40	0.076	12.48	1.56
**Apr**	**2.5%**	6.50	0.04	0.24	0.08					2.83		1.95	0.010	1.69	0.20
	**25%**	13.86	0.08	0.51	0.18					5.80		3.92	0.021	3.38	0.40
	**50%**	20.55	0.13	0.74	0.28					8.32		5.61	0.030	4.87	0.59
	**75%**	30.50	0.20	1.10	0.43					12.09		8.12	0.044	7.01	0.86
	**97.5%**	64.99	0.45	2.25	0.97					24.51		16.05	0.087	14.13	1.80
**May**	**2.5%**			1.41	0.29	0.87	0.09	0.91	0.12	3.14	1.44	1.83	0.007	1.51	0.15
	**25%**			2.96	0.73	1.81	0.27	1.82	0.26	6.24	2.84	3.69	0.016	2.99	0.30
	**50%**			4.37	1.18	2.61	0.44	2.61	0.39	9.00	4.10	5.27	0.024	4.30	0.44

	**75%**			6.41	1.89	3.77	0.69	3.80	0.57	13.01	5.93	7.58	0.035	6.28	0.62
	**97.5%**			13.31	4.35	7.60	1.55	7.53	1.19	26.42	12.03	15.36	0.074	12.68	1.33
**Jun**	**2.5%**	31.96	0.06	0.98	0.10	2.76	0.07	2.78	0.10	3.82		1.81	0.005	1.28	0.05
	**25%**	69.44	0.18	2.01	0.25	5.68	0.28	5.62	0.28	7.76		3.63	0.012	2.56	0.12
	**50%**	101.34	0.32	2.99	0.40	8.14	0.54	8.18	0.48	11.22		5.24	0.018	3.66	0.19
	**75%**	152.11	0.56	4.37	0.64	11.79	0.97	11.71	0.80	16.09		7.58	0.027	5.31	0.29
	**97.5%**	332.45	1.59	9.03	1.53	23.81	2.64	23.61	2.02	32.51		14.77	0.058	10.84	0.65
**Jul**	**2.5%**	30.94	0.11	0.24	0.03	0.91	0.04	0.89	0.03	0.62		1.93	0.006	2.30	0.07
	**25%**	66.69	0.25	0.50	0.06	1.81	0.08	1.83	0.07	1.24		3.93	0.013	4.65	0.17
	**50%**	99.18	0.40	0.72	0.09	2.63	0.13	2.62	0.11	1.80		5.64	0.020	6.68	0.25
	**75%**	147.24	0.63	1.06	0.14	3.80	0.21	3.77	0.18	2.61		8.12	0.030	9.66	0.39
	**97.5%**	308.88	1.50	2.28	0.34	7.69	0.47	7.51	0.43	5.16		16.65	0.064	19.66	0.87
**Aug**	**2.5%**	62.32	0.09	1.19	0.07					1.87	0.51	2.54	0.006	2.71	0.06
	**25%**	133.27	0.30	2.45	0.18					3.71	1.02	5.09	0.014	5.48	0.15
	**50%**	197.97	0.54	3.62	0.30					5.33	1.47	7.30	0.022	7.79	0.24
	**75%**	295.08	0.95	5.31	0.50					7.80	2.16	10.59	0.034	11.28	0.37
	**97.5%**	621.31	2.85	11.07	1.35					15.49	4.30	21.51	0.075	23.19	0.85
**Sep**	**2.5%**	58.64	0.09	2.43	0.24					2.86		1.82	0.005	1.53	0.04
	**25%**	122.98	0.20	5.10	0.65					5.80		3.69	0.012	3.11	0.11
	**50%**	182.91	0.30	7.50	1.09					8.32		5.29	0.019	4.50	0.17
	**75%**	273.92	0.45	11.01	1.81					11.97		7.64	0.028	6.42	0.27
	**97.5%**	589.55	0.98	23.10	4.38					23.92		15.67	0.063	12.84	0.65
**Oct**	**2.5%**	13.04	0.01	1.18	0.32	2.72	0.38	2.66	0.34	0.61	0.18	1.09	0.004	1.22	0.06
	**25%**	26.90	0.02	2.46	0.70	5.49	0.89	5.47	0.74	1.24	0.37	2.22	0.010	2.45	0.13
	**50%**	39.39	0.04	3.59	1.04	7.93	1.36	7.89	1.13	1.80	0.54	3.19	0.014	3.52	0.20
	**75%**	58.39	0.08	5.33	1.56	11.46	2.05	11.41	1.69	2.58	0.77	4.61	0.021	5.12	0.31
	**97.5%**	125.69	0.26	11.00	3.43	23.62	4.44	22.62	3.59	5.32	1.58	9.29	0.044	10.31	0.69

**Nov**	**2.5%**	1.44	0.59					0.95		1.30	0.007	1.42	0.10
	**25%**	3.05	1.26					1.93		2.63	0.014	2.85	0.22
	**50%**	4.46	1.88					2.79		3.81	0.021	4.04	0.33
	**75%**	6.59	2.78					3.99		5.52	0.030	5.87	0.49
	**97.5%**	13.68	5.87					8.16		11.02	0.061	11.66	1.04
**Dec**	**2.5%**	0.24	0.12	0.90	0.24	0.88	0.17	1.53		2.70	0.015	2.92	0.25
	**25%**	0.49	0.25	1.84	0.52	1.81	0.41	3.07		5.37	0.031	5.97	0.54
	**50%**	0.72	0.37	2.65	0.77	2.65	0.62	4.47		7.79	0.045	8.69	0.82
	**75%**	1.06	0.55	3.80	1.14	3.79	0.93	6.49		11.20	0.065	12.61	1.22
	**97.5%**	2.21	1.17	7.87	2.39	7.75	1.98	12.97		22.51	0.131	25.51	2.60

Supplementary Table 12. Monthly exceedance risk (*ER*) estimates for tetracycline genes selection rate (day^{-1}) in water environment of aquaculture ponds (AP) and a river section (RS) within basins at 20, 50, and 80% probabilities subject to OTC residues

Probability	Season	Lanyang		Potzu		Tsengwen		Yenshui		Agongdian		Kaoping		Tungkang	
		AP	RS	AP	RS	AP	RS	AP	RS	AP	RS	AP	RS	AP	RS
**20%**	**Spring**	0.0323	0.0005	0.0092	0.0034	0.0051	0.0011	0.0052	0.0009	0.0241	0.0148	0.0193	0.0002	0.0178	0.0030
	**Summer**	0.0477	0.0031	0.0119	0.0019	0.0156	0.0020	0.0156	0.0016	0.0216	0.0082	0.0214	0.0001	0.0214	0.0015
	**Autumn**	0.0459	0.0008	0.0199	0.0076	0.0129	0.0030	0.0128	0.0025	0.0173	0.0033	0.0167	0.0001	0.0165	0.0015
	**Winter**			0.0070	0.0040	0.0052	0.0017	0.0052	0.0014	0.0118		0.0193	0.0002	0.0210	0.0035
**50%**	**Spring**	0.0293	0.0004	0.0074	0.0024	0.0041	0.0007	0.0041	0.0006	0.0221	0.0129	0.0175	0.0001	0.0160	0.0026
	**Summer**	0.0470	0.0022	0.0102	0.0014	0.0135	0.0012	0.0135	0.0010	0.0194	0.0066	0.0195	0.0001	0.0194	0.0012
	**Autumn**	0.0446	0.0006	0.0177	0.0062	0.0105	0.0022	0.0105	0.0018	0.0154	0.0025	0.0150	0.0001	0.0148	0.0012
	**Winter**			0.0059	0.0033	0.0041	0.0013	0.0041	0.0010	0.0102		0.0174	0.0015	0.0189	0.0029
**80%**	**Spring**	0.0261	0.0003	0.0059	0.0017	0.0032	0.0005	0.0032	0.0005	0.0202	0.0111	0.0158	0.0001	0.0144	0.0022
	**Summer**	0.0462	0.0016	0.0086	0.0010	0.0115	0.0007	0.0115	0.0007	0.0173	0.0052	0.0177	0.0001	0.0175	0.0009
	**Autumn**	0.0430	0.0004	0.0156	0.0051	0.0084	0.0015	0.0085	0.0013	0.0135	0.0020	0.0134	0.0001	0.0132	0.0010
	**Winter**			0.0049	0.0027	0.0032	0.0009	0.0032	0.0007	0.0088		0.0156	0.0001	0.0170	0.0024

Supplementary Table 13. Minimum percent reduction in emission rate of OTC ($P_{\text{Red,min}}$, %) enabling to make the tetR genes selection rate in aquaculture ponds lower than the criterion *E*50 or *BMR*10 at exceedance risk (*ER*) = 0.2, 0.5, and 0.8 during spring, summer, autumn, and winter

Basin	ER	Spring	Summer	Autumn	Winter
				***E*50**	
Lanyang	0.2	42.84	94.11	89.83	
	0.5	26.32	92.60	89.83	
	0.8	5.04	90.71	82.29	
				***BMR*10**	
Lanyang	0.2	93.70	99.35	98.88	
	0.5	91.88	99.18	98.88	
	0.8	89.53	98.98	98.05	
Potzu	0.2	49.68	63.66	82.77	30.85
	0.5	34.56	55.45	79.17	15.06
	0.8	15.07	45.47	74.85	
Tsengwen	0.2	1.19	75.01	67.17	1.50
	0.5		69.16	57.10	
	0.8		61.96	43.89	
Yenshui	0.2	1.63	75.04	66.99	1.51
	0.5		69.14	57.10	
	0.8		61.91	44.26	
Agongdian	0.2	87.73	84.99	78.58	63.41
	0.5	85.65	82.05	74.35	55.92
	0.8	83.23	78.57	69.36	46.97
Kaoping	0.2	81.94	84.72	77.37	81.95
	0.5	78.91	82.15	73.44	78.67
	0.8	75.36	79.15	68.84	74.83
Tungkang	0.2	79.37	84.7	76.92	84.21
	0.5	75.87	82.05	72.97	81.32
	0.8	71.79	78.88	68.36	77.93

Supplementary Table 14. Minimum percent reduction in emission rate of OTC ($P_{\text{Red,min}}$, %) enabling to make the tetR genes selection rate in rivers lower than the criteria *BMR*10 at exceedance risk (*ER*) = 0.2, 0.5, and 0.8 during spring, summer, autumn, and winter

Basin	ER	Spring	Summer	Autumn	Winter
Potzu	0.2			36.80	
	0.5			20.59	
	0.8			0.39	
Agongdian	0.2	72.98	42.30		
	0.5	67.24	24.78		
	0.8	60.27	1.88		

REFERENCES

Baluyut, E. A. (1989). Aquaculture systems and practices: A selected review. Fisheries and Aquaculture Department, Food and Agriculture Organization of the United Nations, Rome, Italy. http://www.fao.org/3/T8598E/T8598E00.htm [Accessed March 23, 2021]

BAPHIQ (Bureau of Animal and Plant Health Inspection and Quarantine, Council of Agriculture, Executive Yuan). (2021). Domestic animal diseases. https://www.baphiq.gov.tw/ws.php?id=4332 [Accessed March 9, 2021]. (in Chinese)

Boyd, C. E., Wood, C. W., Chaney, P. L., and Queiroz, J. F. (2010). Role of aquaculture pond sediments in sequestration of annual global carbon emissions. *Environ. Pollut.* 158, 2537–2540. doi: 10.1016/j.envpol.2010.04.025

Chen, H., Jing, L., Teng, Y., and Wang, J. (2018). Multimedia fate modeling and risk assessment of antibiotics in a water-scare megacity. *J. Hazard. Mater.* 348, 75–83. doi:10.1016/j.jhazmat.2018.01.033

Chen, Y. L., Chen, Y. H., Chan, H. L., Chen, S. H., Lian, S. Y., Lin, W. H., and Yeh, S. R. (2019). The quality surveillance of the post-market veterinary general drugs. *Exp.Rep AHRI* 53, 23–34. https://vettech.nvri.gov.tw/view.php?theme=web_structure&id=2537 (in Chinese)

FACOA (Fisheries Agency, Council of Agriculture, Executive Yuan). (2021). Annual Publication of Fishery Statistics. https://www.fa.gov.tw/cht/PublicationsFishYear/ [Accessed March 9, 2021]. (in Chinese)

Hsieh, H. M., Li, P. H., and Ding, L. (2013). Single variable empirical formula of the river width in the western Taiwan. *J.Taiwan Agricult. Eng.* 59, 49–66. doi: 10.29974/JTAE.201303_59(1).0004 (in Chinese)

Jiménez-Montealegre, R., Verdegem, M., Zamora, J. E., and Verreth, J. (2002). Organic matter sedimentation and resuspension in tilapia (*Oreochromis niloticus*) ponds during a production cycle. *Aquacult. Eng.* 26, 1–12. doi: 10.1016/S0144-8609(01)00086-3

Knapp, C. W., Engemann, C. A., Hanson, M. L., Keen, P. L., Hall, K. J., and Graham, D. W. (2008). Indirect evidence of transposon-mediated selection of antibiotic resistance genes in aquatic systems at low-level oxytetracycline exposures. *Environ. Sci. Technol.* 42, 5348–5353. doi: 10.1021/es703199g

Leal, J. F., Santos, E. B. H., and Esteves, V. I. (2019). Oxytetracycline in intensive aquaculture: water quality during and after its administration, environmental fate, toxicity and bacterial resistance. *Rev. Aquacult.* 11, 1176–1194. doi: 10.1111/raq.12286

Northern Region Water Resources Office. (2014). Safety check of Luo-Dong weir before use: Emergency response plan for dam break. https://www.wranb.gov.tw/media/382205/%E7%BE%85%E6%9D%B1%E6%94%94%E6%B2%B3%E5%A0%B0%E4%BD%BF%E7%94%A8%E5%89%8D%E5%AE%89%E5%85%A8%E8%A4%87%E6%A0%B8%E5%85%8D%E5%82%99%E6%BD%B0%E5%A3%A9%E7%B7%8A%E6%80%A5%E6%87%89%E8%AE%8A%E8%A8%88%E7%95%AB%E8%AA%AA%E6%98%8E%E6%9B%B8.pdf [Accessed March 17, 2021]. (in Chinese)

Steeby, J. A., Hargreaves, J. A., Tucker, C. S., and Kingsbury, S. (2004). Accumulation, organic carbon and dry matter concentration of sediment in commercial channel catfish ponds. *Aquacult. Eng.* 30, 115–126. doi: 10.1016/j.aquaeng.2003.10.001

The 5th River Management Office, WRA. (2005). Report on Current status of Puzih

River. https://ire-123.wrap.gov.tw/integration2017_wrpi_river/FuncModule/SituationSurvey/WebService/public/file/EcologicalSurveyReport/%E7%AC%AC%E4%BA%94%E6%B2%B3%E5%B7%9D%E5%B1%80/94.05_%E6%9C%B4%E5%AD%90%E6%BA%AA%E6%B2%B3%E5%B7%9D%E6%83%85%E5%8B%A2%E8%B3%87%E6%96%99%E5%BD%99%E6%95%B4%E5%A0%B1%E5%91%8A-.pdf [Accessed March 17, 2021]. (in Chinese)

The 7th River Management Office, WRA. (2011). The Feasibility Assessment of Locling the Embankment in Dashu Weir of Kaoping River. https://www.wrasb.gov.tw/Uploads/pi/8%E9%AB%98%E5%B1%8F%E6%BA%AA%E5%A4%A7%E6%A8%B9%E6%94%94%E6%B2%B3%E5%A0%B0%E6%AE%B5%E4%BD%88%E7%BD%AE%E5%A0%A4%E9%98%B2%E5%8F%AF%E8%A1%8C%E6%80%A7%E8%A9%95%E4%BC%B0.pdf [Accessed March 17, 2021]. (in Chinese)

Tsai, C. T. (2006). Influence of reservoir flood releasing on inundation in downstream area (NSC 94-2625-Z-006-002). http://ir.lib.ncku.edu.tw/bitstream/987654321/75006/1/3010700303026.pdf [Accessed March 17, 2021]. (in Chinese)

Water Resources Planning Institute, WRA. (2012). Investigation of Stream Status of Agongdian River. https://ire-123.wrap.gov.tw/integration2017_wrpi_river/FuncModule/SituationSurvey/WebService/public/file/EcologicalSurveyReport/%E7%AC%AC%E5%85%AD%E6%B2%B3%E5%B7%9D%E5%B1%80/101.12_%E9%98%BF%E5%85%AC%E5%BA%97%E6%BA%AA%E6%B2%B3%E7%B3%BB%E6%B2%B3%E5%B7%9D%E6%83%85%E5%8B%A2%E8%AA%BF%E6%9F%A5-.pdf [Accessed March 17, 2021]. (in Chinese)

WRA (Water Resources Agency, MOEA). (2011). The Feasibility Analysis of Utilizing River Bed in Western Taiwan as a Potential Groundwater Recharge Zone. https://books.google.com.tw/books?id=IlgBEAAAQBAJ&pg=SA2-PA58&lpg=SA2-PA58&dq=%E6%9D%B1%E6%B8%AF%E6%BA%AA+%E6%BD%AE%E5%B7%9E%E7%AB%99+%E7%B4%AF%E8%B7%9D&source=bl&ots=PHKeW7Sd4a&sig=ACfU3U0CSm5a2Vcq1qamlU7ofZvf_c9Ktg&hl=zh-TW&sa=X&ved=2ahUKEwjJr_jUsbbvAhVpyosBHdx0D-04ChDoATAEegQIFRAD#v=onepage&q=%E6%9D%B1%E6%B8%AF%E6%BA%AA%20%E6%BD%AE%E5%B7%9E%E7%AB%99%20%E7%B4%AF%E8%B7%9D&f=false [Accessed March 17, 2021]. (in Chinese)

WRA (Water Resources Agency, MOEA). (2015). Hydrological Year Book of Taiwan Republic of China 2015. https://gweb.wra.gov.tw/wrhygis/ebooks/ebook/ebook/hyb2015/default.htm [Accessed February 25, 2021]. (in Chinese)

WRA (Water Resources Agency, MOEA). (2015b). Regulation Master Plan of Yanshui River (Including Tributary Nabalin River). https://www-ws.wra.gov.tw/001/Upload/oldFile//media/58580/%E9%B9%BD%E6%B0%B4%E6%BA%AA%E6%B2%BB%E7%90%86%E8%A8%88%E7%95%AB-%E5%90%AB%E6%94%AF%EF%A7%8A%E9%82%A3%E6%8B%94%EF%A7%B4%E6%BA%AA-1-68.pdf [Accessed March 17, 2021]. (in Chinese)

WRA (Water Resources Agency, MOEA). (2016). Hydrological Year Book of Taiwan Republic of China 2016. https://gweb.wra.gov.tw/wrhygis/ebooks/ebook/ebook/hyb2016/default.htm

[Accessed February 25, 2021]. (in Chinese)

WRA (Water Resources Agency, MOEA). (2017). Hydrological Year Book of Taiwan Republic of China 2017. https://gweb.wra.gov.tw/wrhygis/ebooks/ebook/ebook/hyb2017/default.htm [Accessed February 25, 2021]. (in Chinese)

WRA (Water Resources Agency, MOEA). (2018). Hydrological Year Book of Taiwan Republic of China 2018. https://gweb.wra.gov.tw/wrhygis/ebooks/ebook/ebook/hyb2018/default.htm [Accessed February 25, 2021]. (in Chinese)

WRA (Water Resources Agency, MOEA). (2019). Hydrological Year Book of Taiwan Republic of China 2019. https://gweb.wra.gov.tw/wrhygis/ebooks/ebook/ebook/hyb2019/default.htm [Accessed February 25, 2021]. (in Chinese)

Zhang, Q. Q., Ying, G. G., Pan, C. G., Liu, Y. S., and Zhao, J. L. (2015). Comprehensive evaluation of antibiotics emission and fate in the river basins of China: Source analysis, multimedia modeling, and linkage to bacterial resistance. *Environ. Sci. Technol.* 49, 6772–6782. doi: 10.1021/acs.est.5b00729
